# Acute Myeloid Leukemia(AML) proteins for in silico drug design using pharmacophore modeling, molecular docking and molecular dynamics simulation

**DOI:** 10.1371/journal.pone.0354456

**Published:** 2026-09-02

**Authors:** Partho Bosu, Tonmoy Adhikary, Abu Sayed Rafi, Naveed Iqbal, Fatima Rasool, Payer Ahmed

**Affiliations:** 1 Department of Mathematics, National Institute of Technology, Durgapur, West Bengal, India; 2 Department of Mathematics, Jashore University of Science and Technology, Jashore, Bangladesh; 3 Department of Mathematics, Jagannath University, Dhaka, Bangladesh; 4 Department of Textile Engineering, University of Scholars, Dhaka, Bangladesh; 5 Department of Textile Engineering, Dhaka University of Engineering and Technology, Gazipur, Bangladesh; 6 Department of Textile Engineering, Jashore University of Science and Technology, Jashore, Bangladesh; 7 School of Interdisciplinary Engineering and Sciences, National University of Sciences and Technology, Islamabad, Pakistan; 8 Department of Bioinformatics, The Islamia University of Bahawalpur, Bahawalpur, Pakistan; 9 Chandpur Science and Technology University, Chandpur, Bangladesh; Mayo Clinic Cancer Center, UNITED STATES OF AMERICA

## Abstract

Acute Myeloid Leukemia (AML) is a hematological malignancy characterized by abnormal myeloid cell differentiation, disrupting normal hematopoiesis. This study utilized an integrated computational approach to identify key molecular targets and potential inhibitors for AML therapy. Analysis of the GSE9476 microarray dataset revealed 566 differentially expressed genes (DEGs), including 437 upregulated and 129 downregulated genes. Functional enrichment indicated significant roles in immune response, apoptosis regulation, and hematopoietic pathways. Protein-protein interaction (PPI) network analysis identified ten hub genes—CD44, TNF, IL1B, MYC, STAT1, LCK, CCR7, FCGR3B, MMP9, and CD28—implicated in AML pathogenesis. After examining these ten hub genes’ resolution, R-factor, Diffraction Component Precision Index (DPI), least overfitting criteria, and other crystallographic features, we concluded that LCK and MMP9 were the best options for our future research. Pharmit was used to construct and validate an areceptor ligand pharmacophore model based on the crystal structures of LCK (PDB ID: 6PDJ) and MMP9 (PDB ID: 1GKC). Receptor-based pharmacophore models for LCK and MMP9 with AUC values of 0.79 and 0.85, respectively, were constructed and validated by virtual screening. The filtered compounds underwent ADME/toxicity profiling before being precisely docked in the MMP9 and LCK, which was automated using Python scripts, in order to optimize the screening’s dependability. Among all screened hits, favorable binding affinity values of −9.7 and −9.9 kcal/mol for CID-56715853 and CID-126445459 with LCK and −8.9 and −9.2 kcal/mol for MCULE-1367186142 and ZINC4896454 with MMP9, respectively, showed significant binding interactions. Molecular dynamics analyses, including RMSD, RMSF, PCA, and MM-GBSA, confirmed complex stability and binding affinity. This comprehensive in-silico framework integrates transcriptomic data, network analysis, pharmacophore modeling, and molecular simulations to identify novel biomarkers and drug candidates for AML, providing a foundation for further experimental validation and therapeutic development. These four hits (CID-56715853, CID-126445459 MCULE-1367186142 and ZINC4896454) are potential candidates for further in vitro and in vivo validation.

## 1. Introduction

Acute Myeloid Leukemia (AML) is a severe hematological malignancy marked by the abnormal differentiation of immature myeloid cells, which disrupts normal hematopoiesis [19]. In adults, especially those aged 35–60, the disease commonly manifests as a triad of symptoms: (1) anemia, (2) infection(s), and (3) bleeding, all due to the replacement of normal cells by myeloid blasts [20]. AML arises from genetic alterations in hematopoietic stem cells, with well-defined risk factors that vary among populations, including individuals with Down syndrome, exposure to radiation, or prior chemotoxic treatments [14]. Diagnosis typically involves genetic testing of cells, bone marrow biopsy, and other blood tests. Treatment consists of aggressive chemotherapy doses (such as cytarabine and anthracyclines), with hematopoietic stem cell transplantation being an option in certain cases [42].

The Src family tyrosine-protein kinase Lck (LCK) plays a crucial role in T cell development and activation. LCK interacts with CD4/CD8 co-receptors to phosphorylate ITAMs, initiating T-cell receptor (TCR) signaling that activates ZAP-70 and downstream pathways regulating T-cell function. Altered LCK activity has been associated with immunodeficiencies, autoimmune diseases, lymphoma, and T-cell leukemias [38]. Mutations or overactivity in LCK can lead to inappropriate T-cell responses, while a loss of LCK function may cause immunodeficiencies. Given LCK’s central role in T-cell function, it has emerged as a potential therapeutic target, with inhibitors being explored for both autoimmune diseases and T-cell malignancies [63]. Matrix metalloproteinase-9 (MMP9) is a zinc-dependent enzyme that degrades collagen and gelatin, key components of the extracellular matrix (ECM). MMP9 is vital for tissue remodeling, wound healing, and inflammation. Produced by immune cells such as neutrophils and macrophages, as well as cancer cells, dysregulation of MMP9 has been linked to atherosclerosis, chronic inflammation, and cancer metastasis [44]. MMP9 facilitates tumor invasion and angiogenesis by breaking down ECM components and opening ECM barriers in tumors. It is expressed in response to various mediators, including cytokines, growth factors, and oncogenic signaling pathways. Due to its role in tissue degradation, MMP9 is considered a potential biomarker and therapeutic target, with many researchers working on developing MMP9 inhibitors for treating cancer and inflammatory diseases [44].

Publicly accessible large transcriptome datasets, such as those in the Gene Expression Omnibus (GEO) maintained by NCBI [5], allow researchers to compare gene expression across various studies. High-throughput techniques like RNA sequencing and microarrays have significantly transformed genomics by enabling large-scale investigations into gene expression. These datasets, available to the public, support research into disease mechanisms, biomarkers, and therapeutic targets [73]. In this study, we analyzed differentially expressed genes (DEGs) in acute myeloid leukemia (AML) by reviewing GEO datasets. This allowed us to compare genes in AML patients with those in healthy controls, identifying dysregulated genes that offer valuable insights into AML pathogenesis and treatment options. To link differentially expressed genes to specific biological processes, such as immune response, apoptosis, and cell cycle regulation, researchers have employed advanced bioinformatics methods like pathway enrichment analysis [66]. Integrating these methods not only illuminates the molecular pathways of AML but also identifies potential biomarkers for diagnosis and targets for precision medicine.

The predictive use of pharmacophore modeling supports rational drug design through its ability to identify important structural and chemical features, such as hydrophobic parts and hydrogen bond donors/acceptors, which are required for a molecule to exhibit a given biological activity [75]. Systems such as Pharmit make this sort of drug screening easier through the virtual screening of a chemical database based on a user-defined pharmacophore model [67]. Newer approaches in network pharmacology take available multi-omics data (transcriptome, proteome, etc.) to map disease pathways, potential therapeutic targets, and repurpose drugs, which have been useful for complex diseases such as cancer and neurological disorders [35].

**Fig 1:**
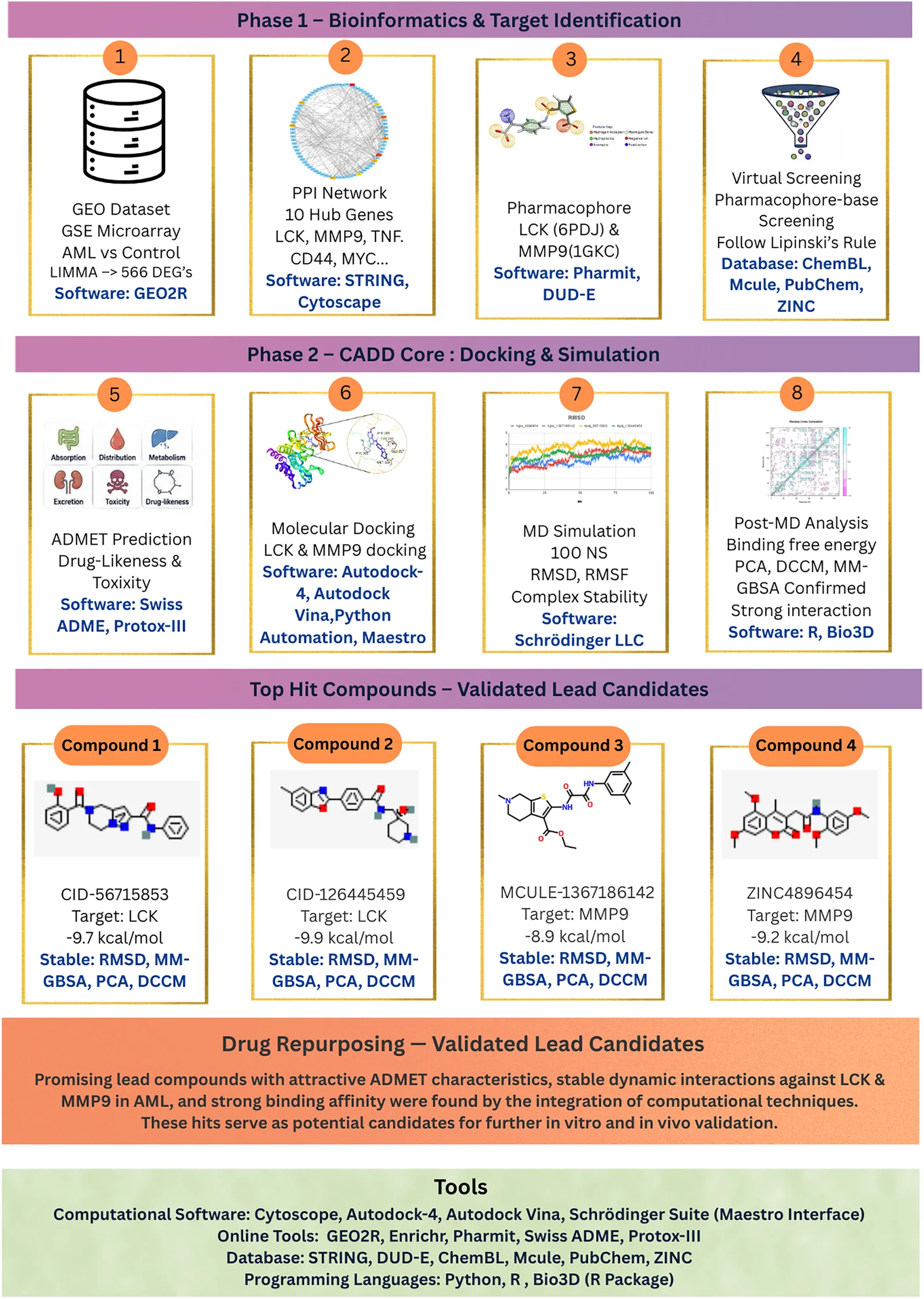
Graphical Abstract.

**Fig 2:**
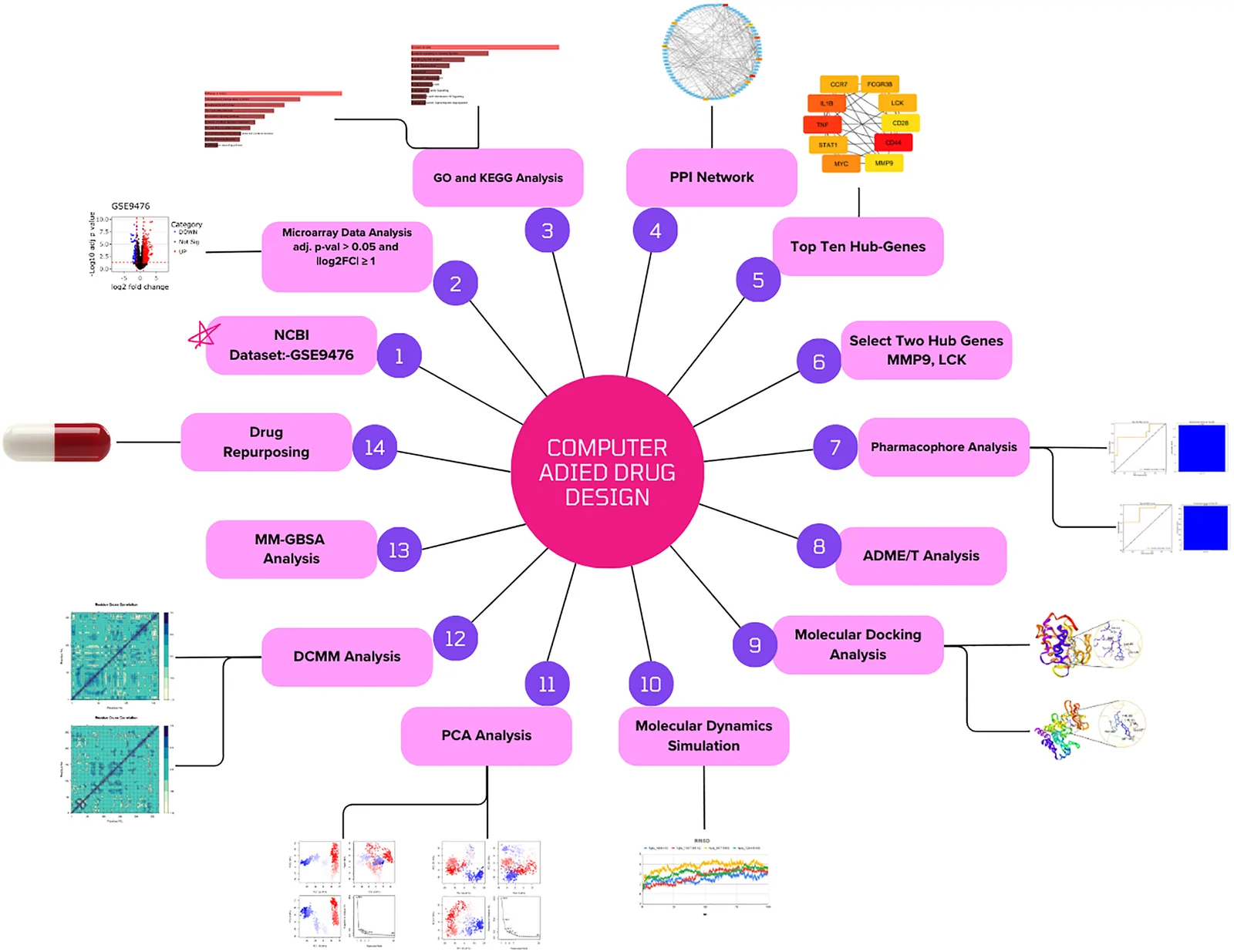
Summary of the findings of this research.

The focus of this study was to perform an integrated analysis of the GSE9476 [65] microarray dataset obtained from GEO to identify differentially expressed genes (DEGs) in acute myeloid leukemia (AML) to identify molecular mechanisms and treatment targets. The approach consists of five key steps: DEG identification of genes found to be significantly upregulated or downregulated in AML when compared with healthy controls; functional enrichment analysis through Gene Ontology (GO) and KEGG analysis of DEGs to gain an understanding of the biological roles and pathways of DEGs; construction and analysis of a protein-protein interaction (PPI) network to allow the identification of hub genes that are important for the underlying pathobiology of AML; pharmacophore modeling with Pharmit to produce a library of drug-like inhibitors of hub genes; and docking and molecular dynamics simulations to assess chemical interactions and binding stability (Fig 1,2). The goal of this study was to identify AML biomarkers and candidate drugs using computational biology.

## 2. Method and materials

### 2.1. Data collection

The datasets used in the study were obtained from the Gene Expression Omnibus (GEO) database maintained by the National Center for Biotechnology Information (NCBI). A gene expression profile with accession number GSE9476 was used [5,65]. The central gene was identified using the GSE9476 dataset, which comprises 64 individuals: 26 patients with AML (cases) and 38 normal hematopoietic samples (controls).

To generate the GSE9476 dataset, scientists at the Fred Hutchinson Cancer Research Center collected and analyzed human acute myeloid leukemia (AML) and healthy control samples in the United States. Therefore, in terms of biological sample origin, it represents a locally collected cohort rather than a multinational or globally sampled population. However, the dataset is publicly available through the Gene Expression Omnibus (GEO), making it widely available and often used for secondary study. GSE9476 is consequently obtained locally but distributed worldwide for research purposes [5,65].

### 2.2. Identification of DEG and common genes

Using the robust multiarray average (RMA) expression metric implemented in the NCBI-GEO2R online tool, the gene expression dataset was first normalized to identify DEGs. DEGs between Acute myeloid leukemia (AML) and control samples were determined using the LIMMA statistical test [26]. Benjamini–Hochberg’s method was applied to control the false discovery rate in multiple testing by adjusting the p-values [7]. Up- and downregulated DEGs were identified based on the adjusted p-value and log2FC thresholds. To select significant DEGs, the cutoff criteria of adjusted *p-value <* 0*.*05, and *|* log2 FC*| ≥* 1 were used.

- **Upregulated DEGs**, if log2 FC *≥*+1*.*00 and adjusted *p*-value < 0*.*05
- **Downregulated DEGs**, if log2 FC *≤−*1*.*00 and adjusted *p*-value < 0*.*05

### 2.3. Protein–protein interaction network analysis to identify hub genes

Various biological functions, such as shape, affinity, and permanence of contact, are influenced by protein interactions, including protein–protein interactions (PPI), which are essential for biological activity [54,74]. Using the STRING database with a confidence score threshold of 700 (high confidence), PPI networks helped identify protein–hub interactions [68]. To detect hub genes, we used Cytoscape software (v.3.10.1), incorporating MCODE and cytoHubba plugins [11,60].

### 2.4. Enrichment analysis of gene sets

The three primary categories of Gene Ontology (GO)—Biological Process (BP), Molecular Function (MF), and Cellular Component (CC)—were used to annotate differentially expressed genes (DEGs). The main driving force behind the development of GO terms is the desire to better understand the cellular processes, molecular mechanisms, and subcellular localization of gene expression [1,13]. The Kyoto Encyclopedia of Genes and Genomes (KEGG) pathway database is a popular tool for interpreting metabolic and other biological pathways, such as signaling, cellular processes, and disease-related pathways, and relies heavily on gene annotation. By mapping DEGs to KEGG pathways, researchers can better understand their functional roles in specific biological contexts and clarify their involvement in metabolic and regulatory networks [40,41]. We used commonly employed research tools, such as Enrichr [47].

### 2.5. Proteins preparations

The Protein Data Bank (PDB) supplied the three-dimensional (3D) structures of two putative receptors, MMP9 (PDB ID: 1GKC) and LCK (PDB ID: 6PDJ) (See Section 4.5, where we described the selection of these two proteins) [8]. Several computational procedures were applied to prepare protein structures for docking. Disulfide bonds were added, bond orders were assigned, and zero-order bonds were created for metal ions. After removing water molecules, the terminal caps were fixed. Prime was used to model the loops and missing side chains. The Schr¨odinger Protein Preparation Wizard was employed for all preparation steps [36,49]. Finally, docking studies were performed with AutoDock Vina using the prepared proteins [50].

### 2.6. Receptor-based pharmacophore

A receptor-based pharmacophore is a model that captures essential structural and chemical features required for biological activity. By examining the receptor’s active site, where ligand binding occurs, it identifies and evaluates the key interactions necessary for molecular recognition and function, such as hydrogen bond donors and acceptors, hydrophobic regions, aromatic rings, and charged centers [75]. Pharmit, an open-source tool for pharmacophore modeling and virtual screening, was used to perform the receptor-based pharmacophore virtual screening. For screening, Pharmit accepts both user-supplied and built-in compound databases. Tyrosine-protein kinase Lck (PDB ID: 6PDJ) and matrix metalloproteinase-9 (PDB ID: 1GKC) were the two protein targets used to generate pharmacophore searches [67].

#### 2.6.1. Binding site analysis.

While analyzing it for pharmacophore modelling, we focused on identifying the binding interactions of the tyrosine-protein kinase Lck (LCK) co-crystallized ligand within the enzyme active site. We used the Protein Plus server to identify significant binding site interactions owing to its efficient features and intuitive design [59]. A similar approach was used for protein matrix metalloproteinase-9 (MMP9).

### 2.7. Pharmacophore validation

**Fig 3:**
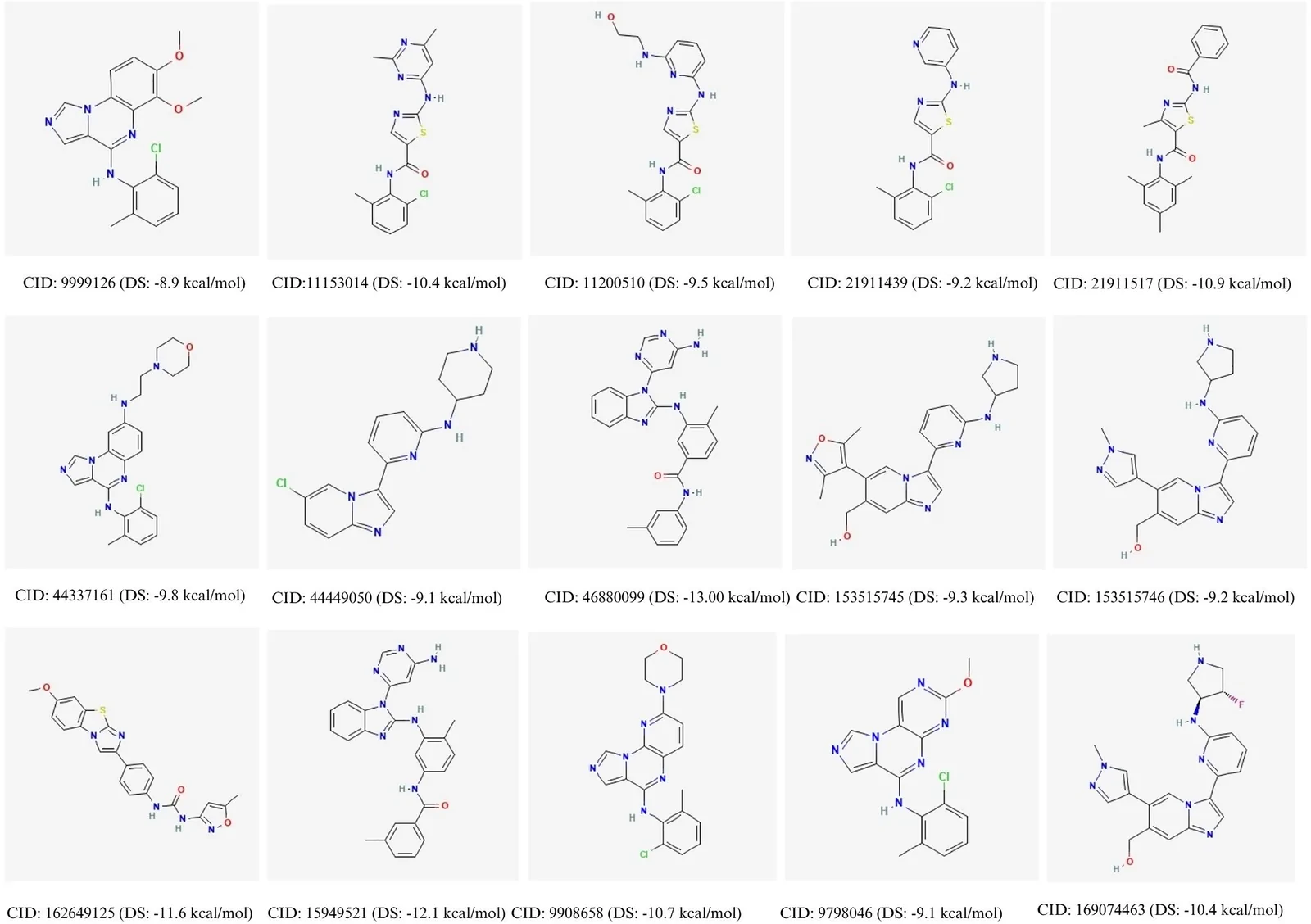
PDB ID: 6PDJ, 15 Active Compounds with CID and DS (Docking score) of LCK Protein.

**Fig 4:**
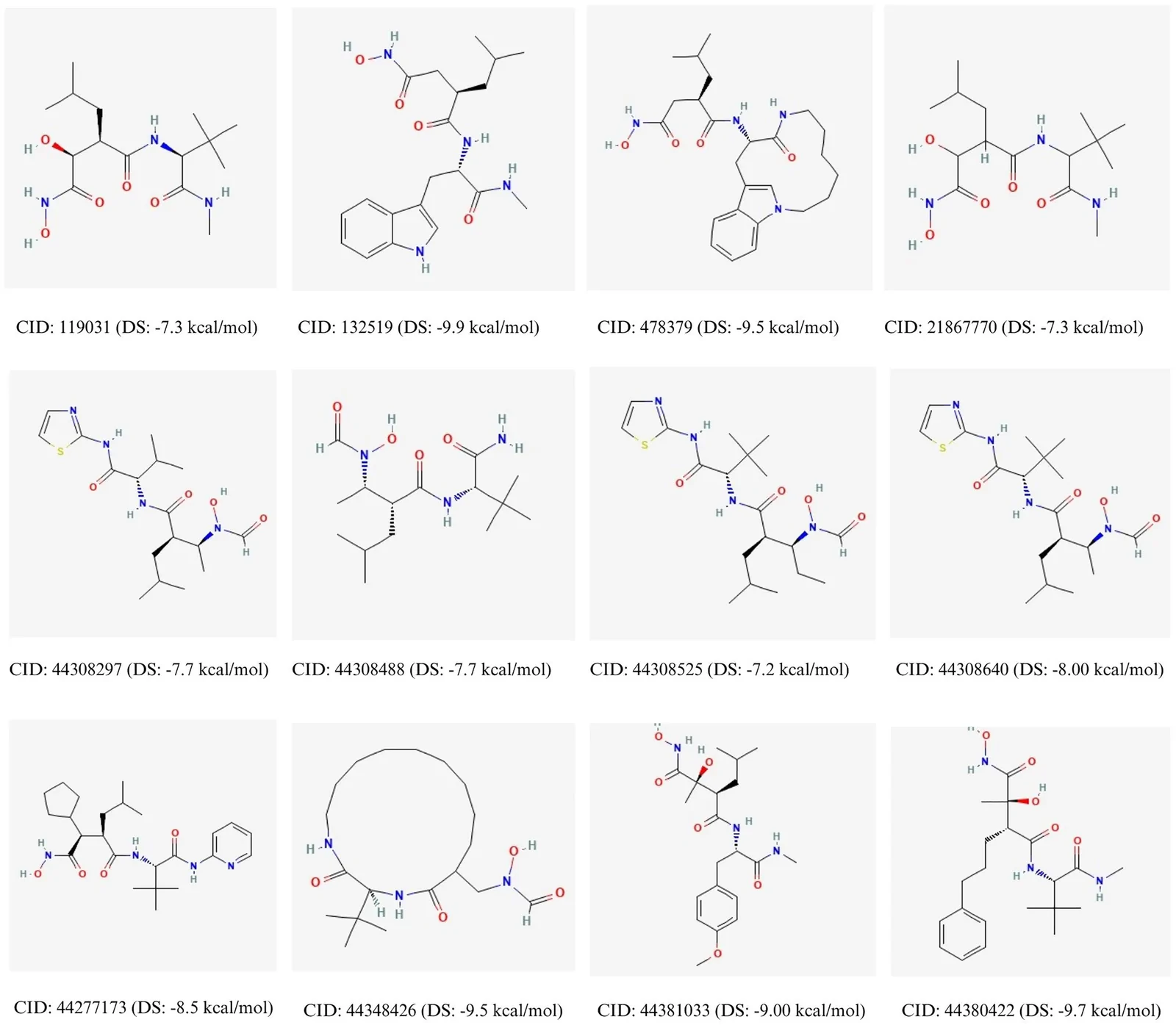
PDB ID: 1GKC, 12 Active Compounds with CID and DS (Docking score) of MMP9 Protein.

Verifying the correctness and dependability of a pharmacophore model is crucial before using it in virtual screening. A collection of known active compounds and a set of inert compounds or decoys specific to the target of interest is required for this validation. The goal of a well-defined pharmacophore model is to detect as many active ligands as possible, while minimizing the detection of inactive compounds or decoys. This was accomplished using a set of 15 chemically synthesized active compounds targeting the tyrosine protein kinase LCK obtained from PubChem (Fig 3). [45]. These active compounds were docked into the binding site of the 6PDJ protein (co-crystallized with tyrosine-protein kinase LCK) using AutoDock Vina [8,69] Similarly, 12 chemically synthesized active inhibitors targeting the MMP9 complex were collected from PubChem (Fig 4) [45]. AutoDock Vina, a well-known molecular docking program, was used to dock these active inhibitors into the binding site of the 1GKC protein (MMP9–inhibitor complex) [8,69]. To validate the pharmacophore model, this step ensured that the active compounds correctly matched the target protein. The **DUD-E** database (http://dude.docking.org/) provides the decoy compounds required for pharmacophore validation. The extensive DUD-E (Directory of Useful Decoys, Enhanced) database includes hundreds of active and decoy compounds for 102 target proteins. Every active ligand generated numerous decoys, guaranteeing a thorough validation procedure. Although these decoys have different 2D topologies, they resemble the physicochemical properties of active ligands (such as molecular weight, solubility, and hydrophobicity) [51]. Decoys are ideal for assessing the specificity and accuracy of pharmacophore models because of their design, which ensures that they are structurally different, but chemically comparable. To identify the model that produced the best results, the active and decoy compounds were uploaded to Pharmit as two separate libraries and tested using the generated pharmacophore models [67,70].

In summary, ChEMBL database was used to extract active compounds that matched the two target proteins (PDB IDs: 1GKC and 6PDJ). 12 active compounds for 1GKC and 15 active compounds for 6PDJ were found. AutoDock Vina was used for molecular docking against the corresponding protein structures in order to verify their binding potential. All of the chosen compounds showed positive interactions with the target proteins, with binding affinities less than −7.0 kcal/mol. Decoy (inactive) compounds were then retrieved for additional validation research from the DUD-E database.

Molecular docking was the primary screening technique utilized to verify the binding potential of active compounds generated via ChEMBL. Pharmacophore modeling was used as an additional technique to identify important interaction features. While docking scores provided quantitative binding affinity estimates, pharmacophore analysis confirmed the presence of critical interaction patterns required for bioactivity.

#### 2.7.1. Receiver Operating Characteristic (ROC) Curve and AUC.

A visual tool for evaluating the effectiveness of a classification model, particularly in machine learning and virtual screening, is the Receiver Operating Characteristic (ROC) curve. It contrasts the True Positive Rate (TPR) with the False Positive Rate (FPR) for various classification criteria [23].

Sensitivity, also known as True Positive Rate (TPR), quantifies the proportion of real activeb substances that were accurately classified as active substances.


$$\begin{array}{c} TPR\;=\;TP/(TP\;+\;FN) \end{array}$$ (1)

The number of inert chemicals that are mistakenly classified as active is known as the false-positive rate (FPR).


$$\begin{array}{c} FPR\;=\;FP/(FP\;+\;TN) \end{array}$$ (2)

In this case, True Positives (TP): Actives that were accurately anticipated. False positives(FP): These are incorrectly anticipated actives that turn dormant. True Negatives (TN): Inactives that were accurately anticipated. False negative (FN): Inactives that were mispredicted but turned out to be active. Area Under the Curve (AUC) The model’s overall capacity to differentiate between active and inactive substances is measured by the area Under the Curve (AUC). AUC = 1.0, perfect classification. AUC = 0.5: Random classification (no predictive ability). AUC *<* 0*.*5: worse than random guessing. [57].

#### 2.7.2. Enrichment analysis.

The Enrichment Factor (EF) is a crucial metric in virtual screening that evaluates the performance of a model better than random selection. It measures the degree to which the screening process prioritizes active compounds within the highest-ranked percentage of compounds [53,61].


$$\begin{array}{c} Mathematical\;expression,\;EFx\;=\;(Ax/Nx)/(Atotal/\;Ntotal) \end{array}$$ (3)

where *Ax* is the number of active compounds in the top *x*% of the rated list. *Nx* represents the number of compounds in the top *x*% of the ranking list. where Atotal is the total number of active compounds in the entire dataset, and Ntotal is the total number of compounds in the dataset. For this study, EF was calculated in the top 3%, indicating the likelihood that an active molecule will appear in the top-ranked proportion compared to random selection [31].

For this study, the EF was calculated in the upper 3%, which indicates the likelihood that an active molecule will be in the top-ranking proportion compared to random selection.

### 2.8. Drug-likeness prediction

A variety of basic molecular characteristics, including molecular weight, number of hydrogen bond donors and acceptors, and octanol/water partition coefficient (AlogP), are important factors in determining whether a chemical is likely to be an oral medication in humans. These traits are frequently predicted using computational methods. These parameters were computed for the hit compounds using the SwissADME platform. Key ADME parameters (absorption, distribution, metabolism, and excretion), physicochemical properties, and other descriptors important to drug-like compounds were calculated using the SwissADME online tool [16]. The chemicals used in this investigation were filtered using Lipinski’s Rule of Five [48]. This rule states that a compound is likely to be orally active if it satisfies the following requirements with no more than one infraction: octanol/water partition coefficient (AlogP) *≤*5, number of hydrogen bond donors (HBDs) *≤*5, number of hydrogen bond acceptors (HBAs) *≤*10, and molecular weight (MW) *≤*500 Da. SwissADME was used to calculate these characteristics, which were then used to filter the hit compounds [72].

### 2.9. ADMET calculation

Drug-likeness and absorption, distribution, metabolism, and excretion (ADME) characteristics are important considerations when assessing possible medicinal compounds for clinical trial advancement because a good drug candidate must have both optimal ADME characteristics and efficacy against a therapeutic target. SwissADME was used to assess drug-likeness and absorption, distribution, metabolism, and excretion (ADME) characteristics of hits. SwissADME is a useful online tool that computes physicochemical characteristics, ADME parameters, and other crucial descriptors necessary to evaluate the potential of drug-like compounds [16]. The prediction of the toxicity of compounds is another crucial step in drug development. The Protox III website was used to assess toxicity endpoints such as cytotoxicity, mutagenicity, carcinogenicity, immunotoxicity, and hepatotoxicity [4].

### 2.10. Molecular docking study

The key steps in drug development include precisely determining the binding free energy of each ligand and determining the ideal binding posture inside the receptor’s binding region. Molecular docking is a popular computer method for predicting how a small molecule (ligand) interacts with a target protein (receptor) and for estimating the intensity of that connection. This study outlines a comprehensive procedure for conducting molecular docking investigations using AutoDock Vina for docking simulations involving multiple ligands and AutoDock-4 for protein preparation [50,69].The docking procedure was automated using a Python script to improve speed and consistency. Maestro software is also used for post-docking analysis, which allows a detailed assessment of the results [24].

### 2.11. Molecular dynamic simulation, PCA and DCCM

We used the Schrodinger LLC Desmond module to perform molecular dynamics simulations to analyze the binding behavior of the top four high-ranked compounds at the atomic level and to gain an understanding of the molecular interactions [2,12]. In an orthorhombic recurrent boundary scenario with dimensions of a 10 °A buffer zone between receptor atoms and box edges, complexes 1GKC 4896454, 1GKC 1367186142, 6PDJ 56715853, and 6PDJ 126445459 were solvated using the TIP3P water model [34]. Neutralization of the solvated technology was achieved by adding 0.15M NaCl combat ions. Subsequently, the system was minimized by using the baseline force field values for OPLS 2005 with a selected 310k and pressure of 1 atm by the NPT ensemble [62]. The data were gathered at intervals of 100 ps and the acquired data were evaluated [62]. The data were gathered at intervals of 100 ps and the acquired data were evaluated [33]. PCA and DCCM were investigated using the R package ”Bio3D” [29].

### 2.12. Molecular mechanics and generalized born surface area calculations

Using the MMGBSA strategy, we were able to determine the impact of the kinetic components on the binding of the ligand-protein complexes. Rotamer search techniques, the OPLS 2005 force field, and the VSGB solvent model were used to drive the binding free energy using the following equation:


$$\begin{array}{c} {\Delta G}_{bind}\;=\;G_{complex}\;-(G_{protein}\;+\;G_{ligand}) \end{array}$$

## 3. Results

### 3.1. Identification of DEGs

**Fig 5:**
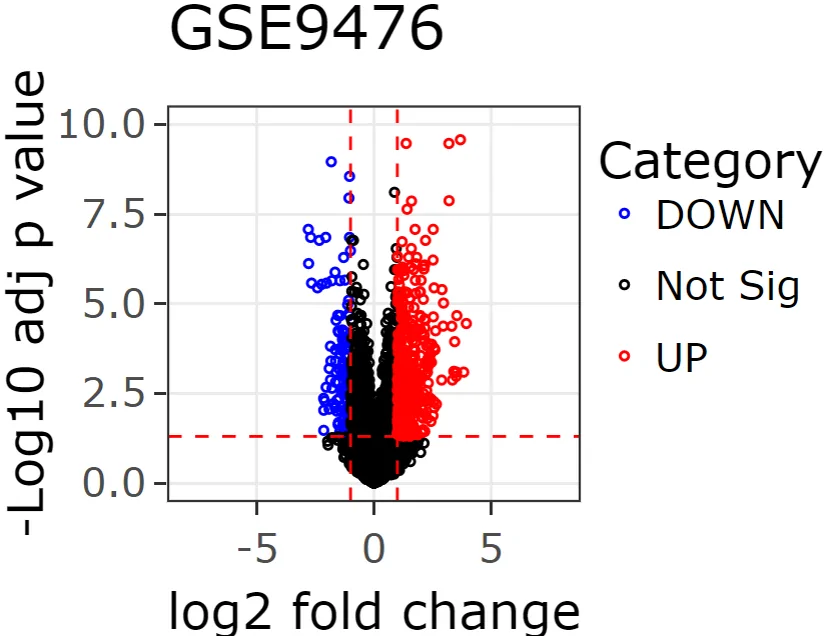
Volcano Plot Showing differentially Expressed Genes (DEGs).

In this investigation, the most current gene expression profile dataset (GSE9476) contained leukemic blasts from 26 patients with acute myeloid leukemia (AML) and samples from 38 healthy donors. Differentially expressed genes (DEGs) were identified by comparing AML samples to normal samples (Table 1). The gene expression patterns of the dataset are represented by a volcano plot (Fig 5) that displays the DEGs.

**Table 1:** Gene expression in the datasets used for microarray data analysis, summarized statistically.

GEO accession	Source	Up-regulated	Down-regulated	Total
GSE9476	Abnormal Expression Changes in AML	437	129	566

Differentially expressed genes (DEG) were selected based on an adjusted p-value < 0.05, and | log2(fold change (FC))| > 1.0. Red dots represent significantly upregulated genes (adjusted p-value < 0.05, log2FC ≥ 1), whereas blue dots indicate significantly downregulated genes (adjusted p-value < 0.05, log2FC ≤−1). DEGs were selected on the basis of predetermined thresholds.

### 3.2. Detection of gene ontology and pathway enrichment analysis

To determine biological importance and identify enriched pathways related to our study, Enrichr was used to perform gene ontology (GO) word and pathway enrichment analysis. The biological roles, cellular mechanisms, and molecular activities of differentially expressed genes (DEGs) were investigated using GO analysis. The table summarizes the top ten enriched phrases in the molecular functions, biological processes, and cellular component categories (Table 2)).

**Table 2:** Conducted Gene Set Enrichment Analysis on the Genes from Microarray Data that Showed Differential Expression in Individuals with AML. Three Tables (A, B, C) Including the Top Ten Rnriched Gene Ontology (GO) Concepts.

A: Biological Process (GO Term).		
Biological Process (GO Term)	P-value	Associated KGs
Cellular Response to Cytokine Stimulus (GO:0071345)	1.03 *×* 10 *−* 9	CEBPA, FOXC1, IFITM2, FLT3, LEF1, CSF2RB, GATA3, IL1RAP, FCAR, ZFP36L1, CRHBP, BAG4, STAT4, CCR7, GBP1, STAT5B, STAT1, IL16, EPOR, EREG, IL1B, IL3RA, IL2RB, XCL1, IL6ST, IL7R, PF4
Cytokine-Mediated Signaling Pathway (GO:0019221)	1.60 *×* 10 *−* 9	CEBPA, FOXC1, FLT3, CSF2RB, IL1RAP, FOXO3, TNF, IL18RAP, STAT4, STAT5B, CCL23, IL15, STAT1, IL16, EPOR, EREG, CEACAM1, IL1B, IL3RA, IL2RB, XCL2, XCL1, IL6ST, IL7R, PF4
Negative Regulation Programmed Cell (GO:0043069)	7.40 *×* 10 *−* 9	NOTCH2, FCMR, LEF1, FHL2, GATA1, FOXO1, IGF1R, PRDX2, BAG4, NUAK2, CCND2, DPP8, GRK5, MYC, NUP62, PRAME, PIM2, SNCA, TIGAR, AREL1, SERPINB9, AZU1, MMP9, WT1, IL2RB, CD28, YME1L1, CD27, IL6ST, CD44
Regulation of Apoptotic Process (GO:0042981)	9.20 *×* 10 *−* 9	NOTCH2, APP, FCMR, FLT3, LEF1, FHL2, FOXO3, GATA1, TNF, FOXO1, IFIT2, IGF1R, PRDX2, BAG4, LGALS1, NUAK2, CCND2, GRK5, MYC, NUP62, PRAME, BRD8, PIM2, PHLDA1, CTSD, SNCA, AREL1, STAT1, BIK, CDKN2A, SERPINB9, AZU1, MMP9, RYBP, BIN1, WT1, IL2RB, CD28, YME1L1, CD27, CALR, IL6ST, CD44
Regulation of Cell Population Proliferation (GO:0042127)	1.49 *×* 10 *−* 8	CEBPA, APP, BTG2, IFITM1, CDKN1B, FLT3, GATA3, IGF1R, ZFP36L1, ADGRG1, CCND2, GRK5, MYC, NAMPT, LMNA, NUP62, STAT4, NCK2, WWC3, PRAME, TIMP1, PIM2, CD33, TPD52, SRPK2, STAT5B, CCL23, JAG1, STAT1, CDKN2A, IL15, CLEC11A, EPOR, EREG, TGFBR2, TIAM1, RGCC, WT1, IL1B, IL3RA, CALR, IL6ST, IL7R, TSPYL5, PF4
Antigen Receptor-Mediated Sig-naling Pathway (GO:0050851)	6.54 *×* 10 *−* 8	ITK, BTK, TNFAIP3, BTG3A, BTNSA3, TRAF, CD79A, ZAP70, LCK, CD28, BLNK, PD4EB4, CD247, MS4A1, SKAP1
Lymphocyte Differentiation (GO:0030098)	1.79 *×* 10 *−* 7	TPD52, ZAP70, CD79A, FLT3, LCK, BLNK, RHOH, DNABJ9, PAX5, GATA3, MS4A1, PAX1
Negative Regulation of Apop-totic Process (GO:0043066)	2.82 *×* 10 *−* 7	NOTCH2, FCMR, LEF1, FHL2, GATA3, GATA1, TNF, FOXO1, IGF1R, PRDX2, BAG4, NUAK2, CCND2, GRK5, MYC, NUP62, PRAME, PIM2, SNCA, PRKCI, AREL1, SERPINB9, AZU1, MMP9, WT1, IL2RB, CD28, YME1L1, CD27, IL6ST, CD44
T Cell Activation (GO:0042110)	4.27 *×* 10 *−* 7	ITK, RHOH, GATA3, TARP, RASGRP1, ZAP70, LCK, CD28, NCK2, CD27, FR1L1, CCR7, CD247, CD44
Positive Regulation of Cellular Process (GO:0048522)	5.72 *×* 10 *−* 7	APP, FLT3, TNF,IGF1R, ZFP36L1, CDCA4, CCND2, GRK5, MYC, SPOCK2, NAMPT, LCK, NCK2, ADGRG1, TIMP1, TPD52, SRPK2, STAT5B, CLEC11A, TPI1, TARP, STAT1, CDKN2A, EPOR, EREG, TGFBR2,TIAM1, RGCC, WT1, IL1B, IL3RA, CALR, IL6ST, IL7R, CCR, TSPYL5
**B:** **Cellular Component (GO Term)**		
**Cellular Component (GO Term)**	**P-value**	**Associated KGs**
Tertiary Granule (GO:0070820)	2.92 *×* 10 *−* 5	MGAM, TNFAIP6, HBB, FPR2, PPBP, MMP9, FCAR, CEACAM1, QPCT, ENPP4, CD59, PTX3, CTSD, CD33
Tertiary Granule Lumen (GO:1904724)	2.45 *×* 10 *−* 4	TNFAIP6, QPCT, HBB, PTX3, PPBP, CTSD, MMP9
Platelet Alpha Granule (GO:0031091)	2.49 *×* 10 *−* 4	APP, SPARC, SERPINE2, ITGA2B, TIMP1, PPBP, F5, SNCA, PF4
T Cell Receptor Complex (GO:0042101)	3.24 *×* 10 *−* 4	ZAP70, CEACAM1, CD247, TARP
Specific Granule (GO:0042581)	3.60 *×* 10 *−* 4	CEACAM1, ANXA3, QPCT, CTSZ, LCN2, CD59, PTX3, FPR2, CTSD, FCAR, CD33, CKAP4
Nuclear Membrane (GO:0031965)	5.14 *×* 10 *−* 4	ENO1, PTGS2, LTC4S, SYNE2, MFSD10, SYNE1, CCND2, GRK5, SCRN1, NUP50, LMNA, NUP62, NUP98, LRIF1, LBR
Membrane Raft (GO:0045121)	6.93 *×* 10 *−* 4	APP, CD79A, ZAP70, LCK, IL6ST, CD24, TNF, CTSD, MS4A1, SKAP1, TGFBR2, ADD2
Secretory Granule Membrane (GO:0030667)	2.42 *×* 10 *−* 3	MGAM, SPARC, ITGA2B, FPR2, AZU1, FCAR, CKAP4, CEACAM1, SCXR1, SELL, CD59, ENPP4, CD33, CD44, SNCA
Ficolin-1-Rich Granule (GO:0101002)	4.01 *×* 10 *−* 3	MGAM, TNFAIP6, PLPPR3, QPCT, CTSZ, HBB, ENPP4, FPR2, CTSD, MMP9, FCAR
Platelet Alpha Granule Lumen (GO:0031093)	4.43 *×* 10 *−* 3	APP, SPARC, TIMP1, PPBP, F5, PF4
**C:** **Molecular Function (GO Term)**		
**Molecular Function (GO Term)**	**P-value**	**Associated KGs**
SH2 Domain Binding(GO:0042169)	3.48 *×* 10 *−* 6	IL18RAP, CXCR1, FLT3, IL3RA, IL2RB, CSF2RB, IL1RAP, IL6ST, IL7R, CD44, EPOR
Protein Kinase Binding (GO:0019901)	1.89 *×* 10 *−* 5	APP, CDKN1B, RNF13, FOXO3, ADD2, CDC42, TCL1A, CCND2, GRK5, PRKAR2B, STK38, BLNK, PRKCH, DUSP3, CDKN2A, MICAL2, RHOH, REXO2, NELL2, CEACAM1, RGC32, LCK, MAPKAPK2, CD247, CD24, CRK, TRIB2, SKAP1, PTP2
Actin Binding (GO:0003779)	4.60 *×* 10 *−* 4	TPM1, MICAL2, SYNE2, MTSS1, SYNE1, ADD2, EPS8, CEACAM1, ABLIM1, MYH10, GBP1, SNCA, MSRB1
Endopeptidase Inhibitor Activity (GO:0004866)	4.60 *×* 10 *−* 4	TPM1, MICAL2, SYNE2, MTSS1, SYNE1, ADD2, EPS8, CEACAM1, ABLIM1, MYH10, GBP1, SNCA, MSRB1
SH2 Domain Binding (GO:0042169)	5.92 *×* 10 *−* 4	LCK, NUP62, BLNK, CRK, SKAP1
Oxidoreductase Activity, Acting on the CH-CH Group of Donors, NAD or NADP as Acceptor (GO:0016628)	8.54 *×* 10 *−* 4	DPYD, BLVRB, LBR, BLVRA
Transcription Cis-Regulatory Region Binding (GO:0000976)	1.16 *×* 10 *−* 3	NOTCH2, CEBPA, FOXC1, GFI1, LEF1, ENO1, GATA3, AHR, FOXO3, GATA1, TNF, BASP1, MYC, SNCA, KCNH2, NFE2, STAT1, ZBTB10, PBX1, KLF1, SETX, SLC25A37, TAL1, WT1, IRF2
DNA-binding Transcription Activator Activity, RNA Polymerase II-specific (GO:0001228)	1.36 *×* 10 *−* 3	NOTCH2, STAT5B, CEBPA, FOXC1, HLF, JDP2, EF1, GATA3, ZNF24, FOXO3, GATA1, FOXO1, HOXA10, GFI1B, WT1, MYC, IRF2, MYBL1
Kinase Binding (GO:0019900)	1.73 *×* 10 *−* 3	CEBPA, CDKN1B, DUSP3, CDKN2A, CD160, RHOH, FOXO3, REXO2, ADD2, CDC42, TIAM1, TCL1A, CEACAM1, RGC32, PRKAR2B, STK38, WWC3, BLNK, CD24, CRK, PFKM, SKAP1
C2H2 Zinc Finger Domain Bind-ing (GO:0070742)	1.80 *×* 10 *−* 3	WT1, LEF1, GATA1

**Fig 6:**
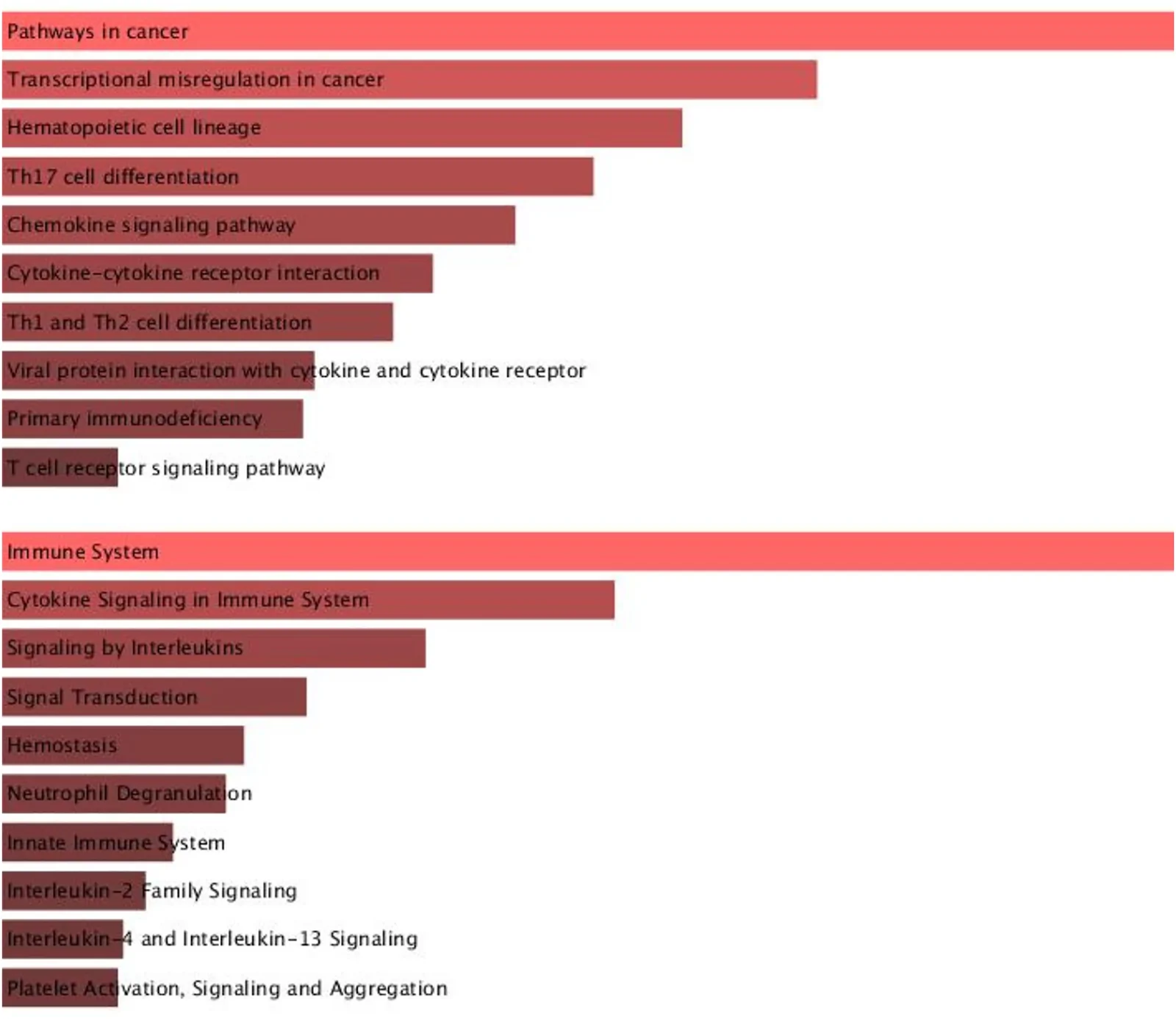
KEGG pathway and reactome pathway.

Functional enrichment analysis was performed to identify the biological pathways associated with the shared differentially expressed genes (DEG). The Kyoto Encyclopedia of Genes and Genomes (KEGG) pathway analysis was used to identify significant pathways such as”Hematopoietic cell lineage,” ”Transcriptional misregulation in cancer,” and ”Pathways in cancer.”The Reactome pathway analysis revealed enrichment for pathways including ”Immune System”and ”Cytokine Signaling in Immune System.” The results of the KEGGand Reactome pathways are shown in Fig 6.

### 3.3. Protein–protein interaction analysis

**Fig 7:**
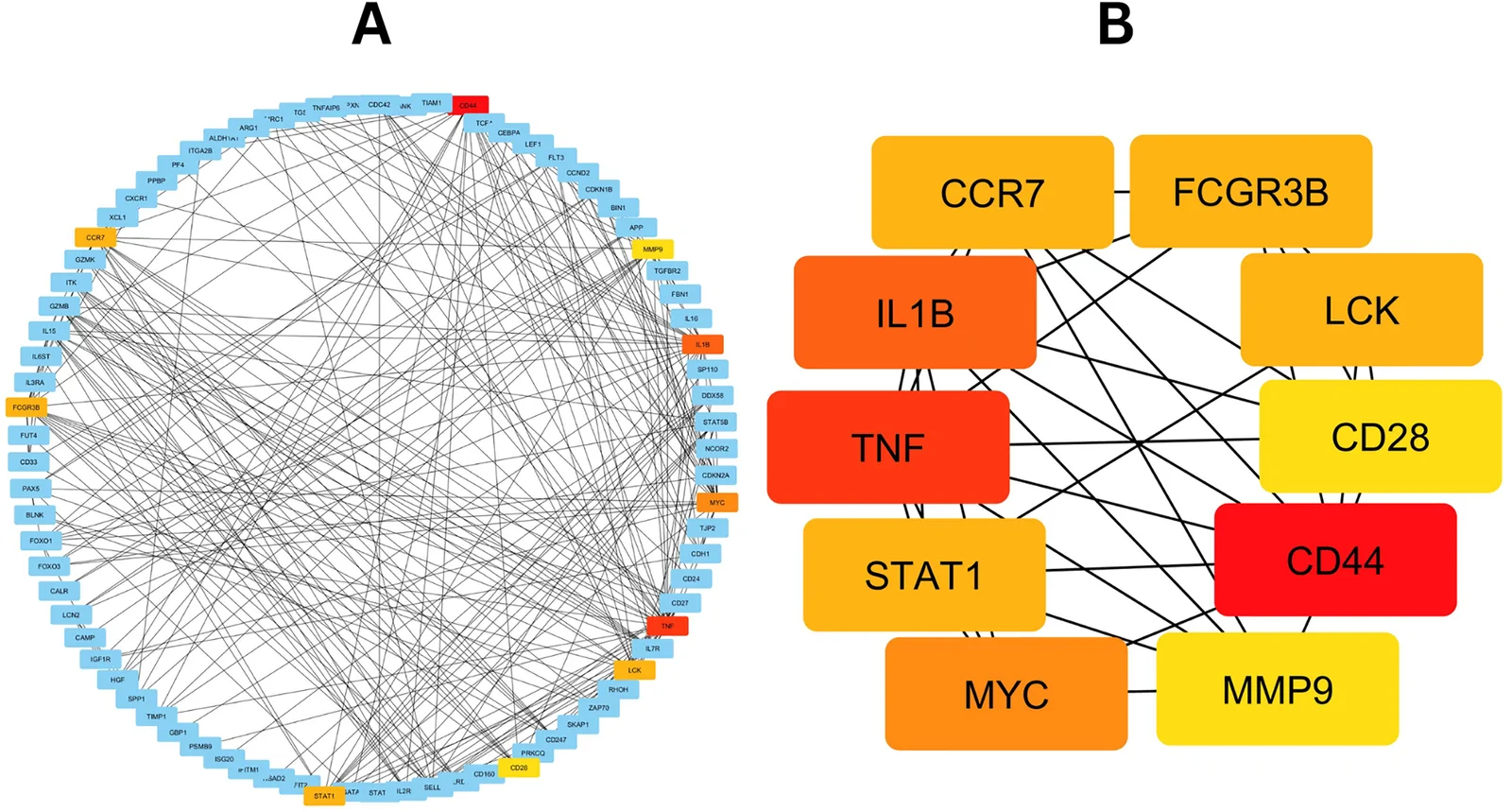
A) Protein–Protein Interactions (PPIs) Network Analysis of DEGs. B)Top 10 Hub Genes Identified by using Cytoscape and CytoHubba.

The visual representation of the protein-protein interaction (PPI) network generated by network analysis revealed important protein centers (Fig 7A). The top ten hub genes are displayed using Cytoscape (v3.10.1), along with the connections between them as established using Network Analyst and Cytoscape (v3.10.1). The PPI network was analyzed to identify protein centers among 566 differentially expressed genes (DEGs). Four different cytoHubba methods were used to identify the key proteins (Fig 7B). These hub proteins, including CD44, TNF, IL1B, MYC, STAT1, LCK, CCR7, FCGR3B, MMP9, and CD28, were identified using MCC, DMNC, Degree, and EPC methods.

### 3.4. Individual Significance of the 10 Hub Proteins in AML

The ten hub proteins that our protein-protein interaction (PPI) network analysis revealed CD44, TNF, IL1B, MYC, STAT1, LCK, CCR7, FCGR3B, MMP9, and CD28 were not chosen at random; as has been widely documented in the peer-reviewed literature, each has established roles in AML pathogenesis, disease progression, immune modulation, and therapeutic targeting. Below is a detailed description of each of their particular significance.

**Cluster of Differentiation 44(CD44):** The primary cellular adhesion molecule that mediates contacts between AML cells and the bone marrow stromal niche is CD44, which is a type I transmembrane glycoprotein. It has a direct role in the development Operationof leukemic stem cells (LSCs) in AML. AML susceptibility has been strongly linked to functional SNPs in the CD44 gene, namely the rs13347C>T variation in the 3’UTR. TT carriers had an approximately 2.67-fold higher risk than CC carriers. Crucially, repeated transplantation trials verified direct targeting of AML LSCs, and in vivo treatment of an activating anti-CD44 monoclonal antibody significantly decreased leukemic repopulation in NOD-SCID mice transplanted with human AML. By disrupting LSC homing to stem cell-supportive niches and changing LSC destiny, the process establishes CD44 as a crucial regulator of AML stemness and a proven therapeutic target [37,81].

**Tumor Necrosis Factor(TNF):** Pro-inflammatory cytokine that is crucial to the pathophysiology of AML is tumor necrosis factor alpha (TNF-α). The c-Jun N-terminal kinase (JNK) pathway, which has Longevity and Growth signaling cascade that functions in tandem with NF-κB in AML stem cells and that is activated by TNF. That is autocrinally generated mainly by AML LSCs and leukemic progenitors. High TNF levels are linked to aggressive illness and poor clinical outcomes in human AML. It was discovered that TNF receptors (TNFR1 and TNFR2) were required to maintain the proliferative advantage of mutant clones in preclinical animal models including mutant DNMT3A hematopoietic stem and progenitor cells.Crucially, co-suppression of TNF and IL-1β signaling works in concert with NF-κB inhibition to eradicate AML LSCs in vivo and ex vivo, with M4/M5 AML subtypes showing the strongest effects. TNF is a therapeutically useful target for removing the AML stem cell compartment [82–84].

**Interleukin 1 Beta (IL1B):** One important pro-inflammatory cytokine is IL-1β and it promotes the development of AML and treatment resistance through abnormal signaling pathways. The NLRP3 inflammasome, which requires caspase-1 to activate IL-1β and it is significantly expressed and activated in AML bone marrow leukemia cells. Its increased activity is strongly associated with a poor outcome in AML patients. Leukemia cell proliferation is enhanced, apoptosis is inhibited and resistance to conventional treatment is increased by IL-1β-dependent activation of NLRP3. Leukemic cell Growth is stimulated by exogenous IL-1β, while leukemia development is suppressed by IL-1β knockdown or anti-IL-1β antibody therapy. IL-1β further speeds up tumor development by encouraging the production of AML growth factors such GM-CSF, G-CSF, and IL-6. While low IL-1β and high IL-1 receptor antagonist (IL-1RA) levels indicate more positive responses, higher IL-1β levels in AML patients predict poorer clinical outcomes. All of these findings point to IL-1β as a crucial mediator in the biology of AML and a logical target for treatment [85–87].

**MYC Proto-Oncogene (MYC):** The MYC proto-oncogene contains a master transcriptional regulator that controls cell growth, metabolism, apoptosis, and differentiation. MYC is frequently overexpressed in AML due to pathways such trisomy 8, FLT3-ITD signaling, gene amplification, and lncRNA-mediated post-transcriptional regulation. Its dysregulation is thought to be one of the crucial oncogenic processes necessary for leukemogenesis. In AML models based on mice shRNA-mediated MYC knockdown significantly increases survival, indicating MYC’s crucial role in disease maintenance. MYC functions downstream of a number of carcinogenic pathways, including BET protein-mediated transcription, JAK/STAT, and PI3K/AKT/mTOR signaling. It is consequently considered a potential therapeutic target with several indirect targeting strategies, such as BET inhibitors (like JQ1), aurora kinase inhibitors (like VX-680), and PI3K inhibitors. MYC have shown preclinical effectiveness in MYC-overexpressing AML [88–90].

**Signal Transducer and Activator of Transcription 1 (STAT1):** The limitation of AML myeloid differentiation is mostly dependent on Signal Transducer and Activator of Transcription 1 (STAT1). AML is characterized by a halt in differentiation, where myeloid development depends on STAT1. Dasatinib-induced differentiation is associated with phosphorylation of STAT1 at Tyr701 and Ser727 and its translocation from the cytoplasm to the nucleus. The transcription of STAT1-target differentiation genes is subsequently encouraged by this activation. STAT1 knockdown by shRNA significantly reduced dasatinib-induced differentiation in AML cells. This indicates that STAT1 activity is necessary for proper myeloid maturation.It was shown that MEK/ERK kinases controlled this activation upstream, and MEK inhibitors prevented both myeloid differentiation and STAT1 phosphorylation. STAT1 represents a molecular target for differentiation-based AML treatments and a functionally important regulatory node in AML differentiation programs [91].

**Tyrosine-protein kinase Lck (LCK):** LCK refers to a class of non-receptor tyrosine kinases called Src-family kinases (SFKs). LYN, FYN, HCK, and FGR are also members of this family.These kinases have all been directly linked to dysregulated AML signaling pathways. SFK-mediated signaling is implicated in the abnormal proliferation, differentiation block, and survival of AML cells. The LCK hub protein in our PPI network suggests its functional interconnectedness within the larger AML kinase signaling landscape. The clinical efficacy of tyrosine kinase inhibitors (TKIs) in AML including quizartinib, gilteritinib, and midostaurin has established the biological tractability of kinase-targeted strategies. LCK as a hub SFK is a good candidate for further inhibitor development in the context of T-cell mediated anti-tumor immunity where LCK is crucial for T-cell receptor (TCR) signal transduction.Its possible involvement in leukemic cell signaling and in regulating the AML immune milieu is reflected in its inclusion in our AML PPI hub network [92,93].

**C-C Chemokine Receptor Type 7(CCR7):** The tumor suppressor gene C-C Chemokine Receptor 7 (CCR7) has an important impact on the AML tumor immune microenvironment (TIME). AML samples express CCR7 at much lower levels than normal controls.

This finding is supported by bioinformatics analysis of AML transcriptome datasets (GSE9476 and GSE114868).After FLT3, CCR7 exhibited the second-highest ROC curve AUC among the hub genes. Strong diagnostic performance in differentiating AML samples from controls is shown by this. CCR7 expression has a substantial positive link with immunological, stromal, and ESTIMATE scores in AML, according to the application of the ESTIMATE methodology. CCR7 is an important regulator of immune cell invasion and TIME remodeling. Normal T-cell and dendritic cell homing, CCR7 suppression in AML may aid in immune evasion. These results indicate the significance of CCR7 as a potential target for immunotherapy in AML as well as a biomarker for diagnosis and prognosis [94].

**Low affinity immunoglobulin gamma Fc region receptor III-B (FCGR3B):** FCGR3B produces the GPI-anchored Fc gamma receptor CD16b, which is mostly expressed on neutrophils. One of the innate immune effector functions it participates in is antibody-dependent cellular cytotoxicity (ADCC). Fc receptor mediated signaling has an important role in immune surveillance and anti-tumor cytotoxicity. In AML, the dysregulation of Fc receptor pathways impairs innate immune effector function, allowing leukemic cells to escape immune surveillance. The identification of FCGR3B as a hub in the PPI network emphasizes the innate immune axis in AML pathobiology. This highlights an important feature of disease biology that is increasingly being linked to response to therapy.It is particularly important for antibody-based AML therapies like gemtuzumab ozogamicin and CD47-targeting medications that depend on Fc receptor-mediated cytotoxicity. FCGR3B also draws attention to possible interactions between the other hub proteins in the AML interaction network and innate immune effector pathways [98,99].

**Matrix Metallopeptidase 9(MMP9):** Matrix Metalloproteinase-9 (MMP9) is a zinc-binding metalloproteinase that acts by breaking down the extracellular matrix elements such as collagen type IV and elastin. This results in increased ability of the leukemic cells to invade, migrate, and remodel the bone marrow microenvironment. MMP9 has been identified to be highly expressed in AML cells and plays a role in the maintenance of cancer stem cells. Experimental models using the broad spectrum MMP inhibitor prinomastat (AG3340) have shown decreased progression of MLL-AF9 AML in animals. This is believed to occur due to improved chemotherapy effectiveness and survival of healthy hematopoietic stem cells resulting from the inhibition of MMP9. Increased MMP9 expression has been observed in DC-CIK cells from AML patients used in immunotherapy treatment. The use of MMP9 inhibitors improves the therapy through activation of T cells, induction of cytotoxic effects on the leukemic cells, and apoptosis of the leukemic cells. This makes MMP9 an appropriate candidate for molecular docking and molecular dynamic simulations [95,96].

**Cluster of Differentiation 28 (CD28):** Complete T cell activation and the effectiveness of the antitumor immune response depend on the costimulatory molecule CD28. Often T-cells demonstrate high tiredness, especially in case of CD28 deficiency, resulting in the inability of such cells to combat the growth of malignancies. It was found that the number of CD28 negative tired T cells had a high correlation with the development of acute myeloid leukemia (AML). An elevated fraction of CD28 negative TIGIT positive CD8 + T cells in AML patients correlated with poor therapeutic effects. Such individuals had low leukemic-free survival, showing the impact of exhaustion in T cells on AML development. With the achievement of remission, the level of T cell tiredness decreased significantly, especially in cases of CD28 positive T cell subpopulations. Thus, one can see the high degree of association of CD28 expression and AML condition. To achieve the effectiveness of anti-PD-1 treatment, CD28-mediated costimulation is needed within the tumor microenvironment. As can be seen, CD28 plays a significant role in determining the effectiveness of checkpoint immunotherapy in AML patients. CD28 is an important node in the network of AML immune interactions [97].

### 3.5. Protein selection

A protein structure was deemed suitable for docking research if it satisfied the following quality standards: The resolution of the crystal is less than 2.5 Å, R-factor ≤ 0.25, R free is below 0.30, Diffraction-component precision index (DPI) < 0.5 Å, and the least overfitting criteria is (R free − R factor) < 0.05. In addition to crystallographic features, further structural requirements included: (1) a section of the active site free of steric conflicts; (2) complete ligand occupancy; and (3) suitable stereochemistry free of odd bond lengths or angles. The selected structures did not contain any point mutations [78,79].

Pharmacophore models, which require at least three crucial protein-ligand interactions to be deemed valid, were created using the Pharmit web server. However, our analysis revealed an absence of the requisite interactions between the proteins and their cognate ligands [67,80].

#### 3.5.1. Hub gene screening results.

Each hub gene was individually evaluated against all aforementioned criteria using its corresponding PDB structure(s). Of the ten hub genes examined, only two met all specifications:

- LCK HUMAN (UniProt Accession: P06239) [39]
- MMP9 HUMAN (UniProt Accession: P14780) [39]

The remaining eight hub genes failed to meet the criteria due to various structural or quality-related limitations (see Table 3).

**Table 3:** Structural Quality Screening of 10 Hub Genes.

No.	Hub Gene	UniProt ID	PDB ID(s) Examined	Status	Primary Reason for Selection/Exclusion
1	CD44	P16070	4PZ3, 4PZ4, 5BZJ, 5BZN, 5BZS, 5BZR, 2I83, 1UUH	FAILED	Domain-only structures; linker region unresolved. Pharmacophore requires ≥3 interaction points—CD44-ligand complexes show insufficient critical interactions
2	TNF	P01375	1TNF, 2TNF, 2AZ5, 4G3Y, 5FLS, 6GJK, 7JRA	FAILED	Trimeric cytokine with flat, shallow binding surface; lacks suitable small molecule binding pockets for pharmacophore modeling
3	IL1B	P01584	1I1B, 1HIB, 1ITB, 4DEP, 4G6J, 4G6M, 2NVH	FAILED	Beta-barrel structure with limited druggable pockets; active site deeply buried, unsuitable for pharmacophore feature definition
4	MYC	P01106	1A93, 6A5W, 6A5X, 6A5Y, 6A5Z, 2A93	FAILED	Intrinsically disordered protein (IDP)—no stable 3D structure available. Transcription factor devoid of classical ligand binding pockets
5	STAT1	P42224	1YVL, 1BF5, 4N6J, 5D3J, 6N3J, 6VWX	FAILED	Resolution 3.0 Å exceeds 2.5 Å threshold; high flexibility in connecting regions; poor stereochemistry for reliable docking
6	LCK	P06239	6PDJ	PASSED	Meets all criteria: resolution <2.5 Å, R-free <0.30, well-defined ATP-binding site, complete ligand occupancy, ≥ 3 pharmacophore features identifiable
7	CCR7	P32248	6QZH, 7F1R, 7F1S, 7F1T, 8H7O	FAILED	GPCR structure requires Sialidase NanA fusion protein for crystallization; non-native conformation makes pharmacophore modeling unreliable
8	FCGR3B	O75015	6EAQ, 6EAT, 6EAV, 3SGJ, 3SLE, 4W4O, 4W4P	FAILED	Structure in complex with Fc fragment; heavily glycosylated and immune-complexed—not suitable for small molecule pharmacophore development
9	MMP9	P14780	1GKC	PASSED	Meets all criteria: resolution <2.5 Å, R-free <0.30, well-defined catalytic zinc site with extensive inhibitor co-crystal structures for pharmacophore validation
10	CD28	P10747	1YJD, 3T2D, 5J89, 5J8A, 5J8B, 5J8C, 5J8D	FAILED	Resolution 2.7 Å exceeds threshold; antibody Fab complex obscures binding site; R-free approaches 0.30 limit

### 3.6. Pharmacophore modeling

#### 3.6.1. Interactions between LCK and MMP9 Protein with their Ligands.

**Fig 8:**
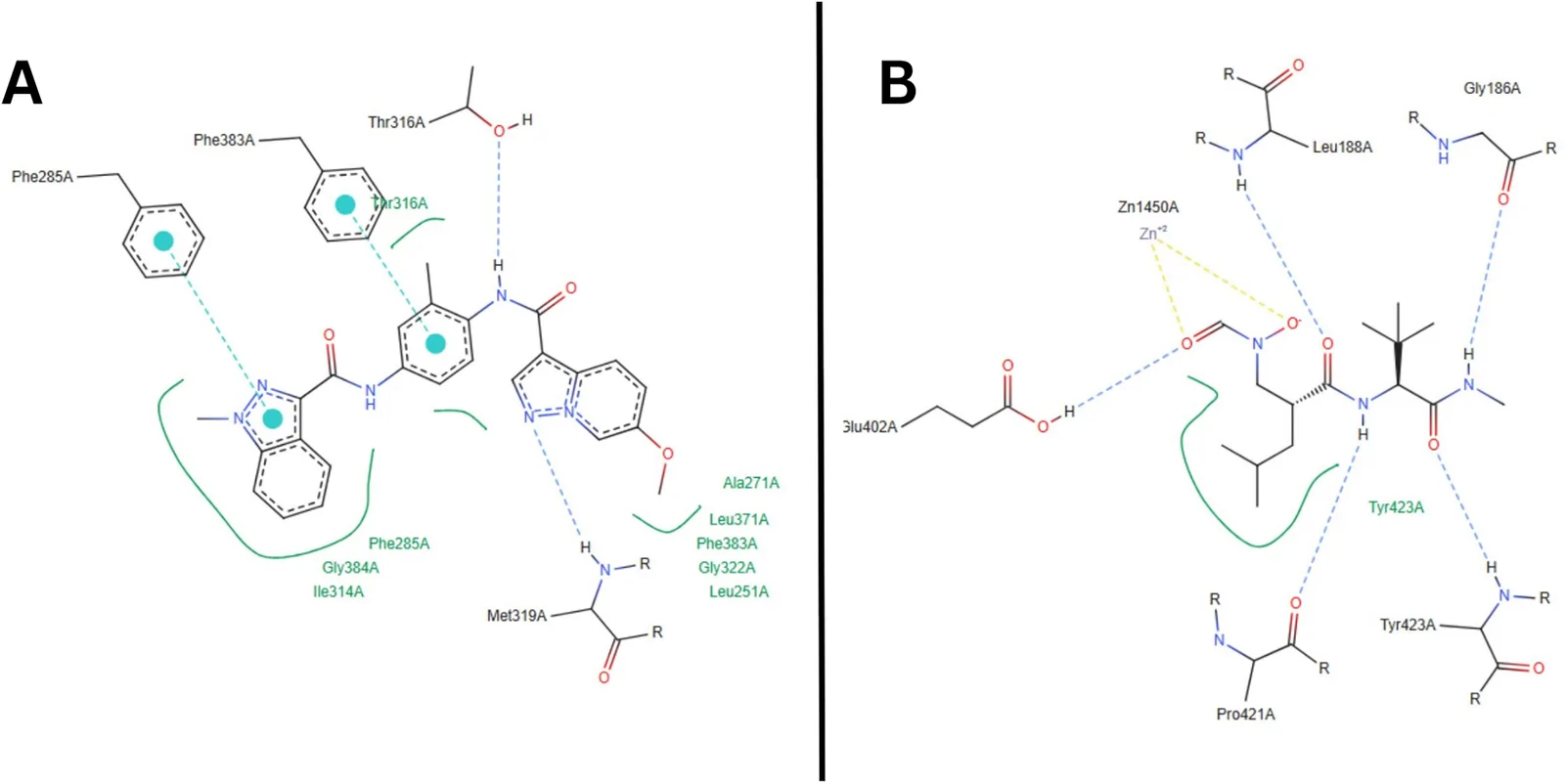
Binging Side of A) LCK Protein (PDB ID: 6PDJ) and B) MMP9 Protein (PDB ID:1GKC).

The LCK protein-ligand complex was shown to have four important interactions: *π*-*π* contact between the ligand’s benzene ring and the side chain of Phe285. Another interaction between *π, π, and* Phe383. Phe383, in turn, forms a hydrogen bond donor relationship, whereas a hydrogen bond acceptor connection is formed by the ligand and hydrogen backbone of Met318. Two *π-π stacking* interactions, one hydrogen bond acceptor and one hydrogen bond donor, are necessary for the stability of the ligand in the active site of tyrosine protein kinase LCK. Because the active site is mainly covered with hydrophobic residues, it is essential to include both hydrophobic and aromatic properties at locations implicated in *π–π* interactions. Thus, two aromatic characteristics (π–π *interactions* with Phe285 and Phe383) were included in the pharmacophore model produced in this investigation. This hydrophobic environment is supported by two hydrophobic characteristics. One acceptor is characteristic of hydrogen bonds (from the Met318 interaction). One characteristic is the donation of hydrogen bonds (from Phe383 interaction) (Fig 8).

**Fig 9:**
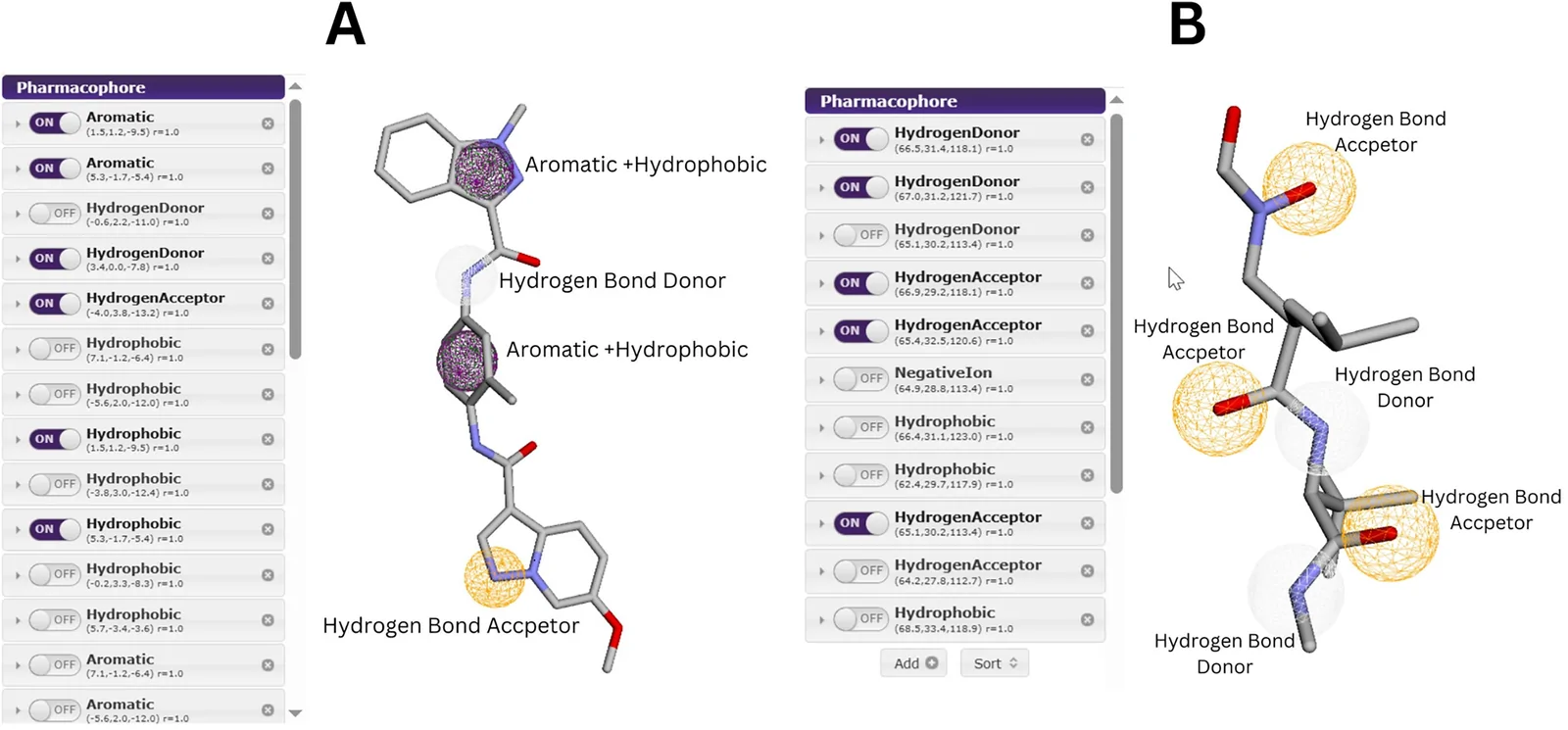
The Structure of the Protein A) 6PDJ and B) 1GKC with their Hydrogen Bond Acceptor, Hydrogen Bond Donor, Aromatics and Hydrophobic Region.

Six pharmacophore characteristics were suggested, guaranteeing the best binding affinity and selectivity for LCK inhibition (Fig 9). Prioritizing these interactions for drug design requires understanding the physicochemical characteristics of the active site.

The MMP9-ligand complex has six important interactions: Zn2+and the ligand form a metal coordination bond (ZN1450). The three interactions with hydrogen bond acceptors were Tyr423, Leu188, and Gly186. Glu402 and Pro421 interact with two hydrogen bond donors. For ligand binding to occur at the MMP9 active site, three interactions are necessary: two hydrogen bond donors, three hydrogen bond acceptors, and coordination. Because metal coordination is typically ignored in pharmacophore modelling, this interaction was excluded from the pharmacophore feature set. As a result, the constructed pharmacophore model included the following: hydrogen bond acceptors have three properties (interacting with Tyr423, Leu188, and Gly186). Two properties (Pro421 and Glu402) work together to create hydrogen-bond donors (Fig 9A. LCK Protein (PDB ID: 6PDJ) & B. MMP9 Protein (PDB ID:1GKC)).

Five pharmacophore characteristics were determined from the MMP9 ligand interaction in this study; due to modeling restrictions, metal coordination was excluded and hydrogen bonding was selected (Fig 9A. 6PDJ & B. 1GKC).

#### 3.6.2. ROC curve and enrichment factor for pharmacophore modelling.

We built a virtual screening database for the LCK protein (PDB ID: 6PDJ) that included 750 carefully matched decoy molecules that were taken from the DUD-E (Directory of Useful Decoys, Enhanced) database and 15 experimentally validated active small molecules. This yielded a final screening library of 765 compounds. In accordance with accepted virtual screening benchmarking techniques, decoy molecules were chosen based on matching molecular weight and comparable physical characteristics, while maintaining topological dissimilarity with the active compounds. In order to facilitate effective 3D similarity-based virtual screening while preserving statistical rigor and biological relevance for LCK kinase inhibitor discovery, this 1:50 active-to-decoy ratio database was created using standard cheminformatics protocols (such as neutralization and tautomer generation) and implemented in Pharmit.

The Receiver Operating Characteristic (ROC) curve for the top 3% of compounds is shown in the Fig, showing the False Positive Rate (FPR, 1-Specificity), which ranges from 0.0 to 1.0, is the X-axis. The sensitivity and True Positive Rate (TPR) on the Y-axis range from 0.0 1.0. The performance of the model is shown by the solid orange curve, which shows a rapid initial rise, suggesting a high capacity for early enrichment. Above the diagonal, the performance was consistent (AUC = 0.79), and the curve became closer to the optimal top-left corner. The random classification performance is indicated by the dashed diagonal line (AUC = 0.5), which acts as a reference point. For the model, the area under the curve (AUC) of 0.79 for the model means that there is a 79% chance that active chemicals will be ranked higher than inactive ones. Strong discriminating ability for applications involving virtual screening.

**Fig 10:**
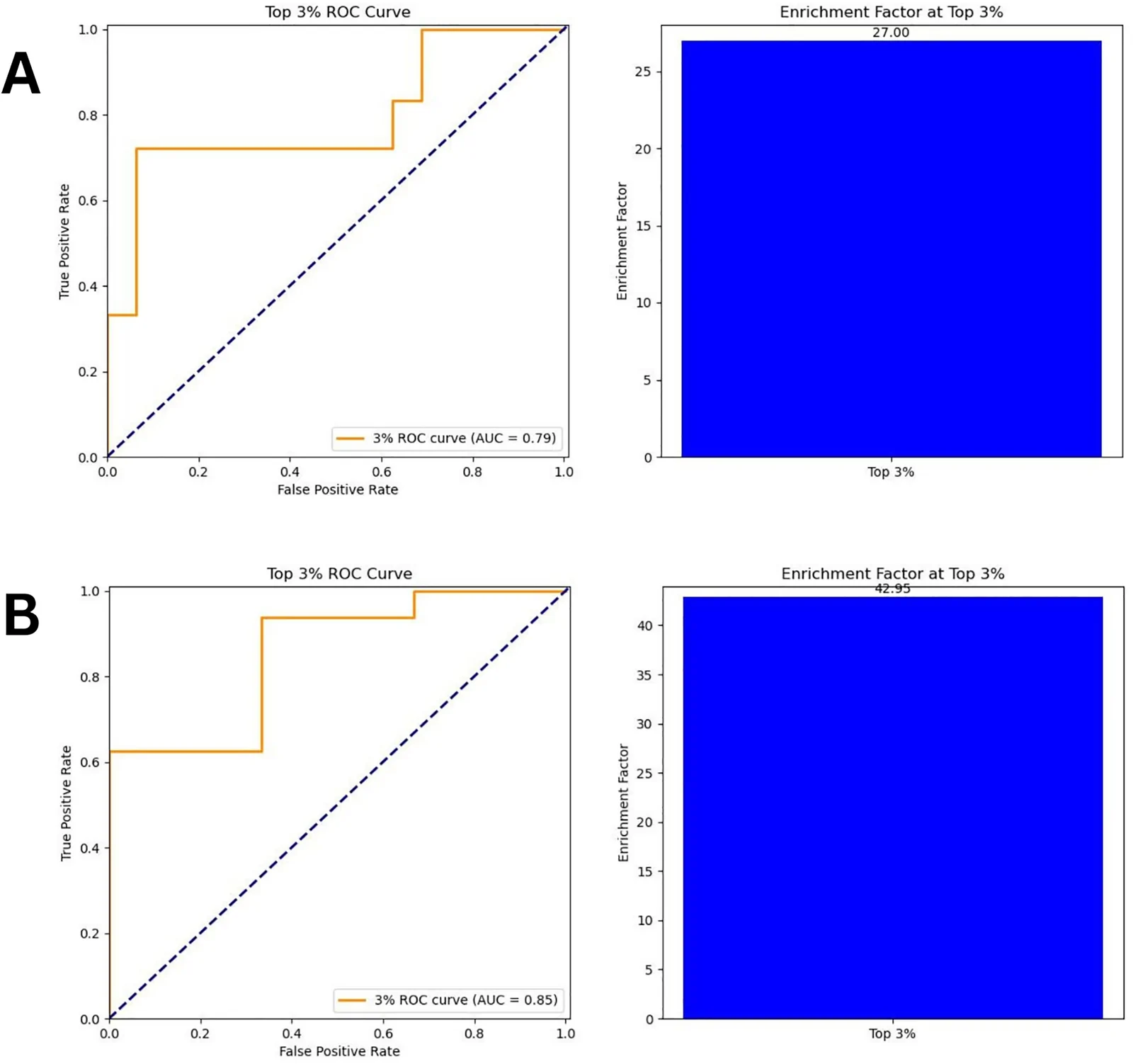
A) ROC curve and Enrichment Analysis for LCK(PDB ID: 6PDJ) Protein. B) ROC curve and Enrichment Analysis for MMP9 (PDB ID: 1GKC) Protein.

The model’s early enrichment capabilities are demonstrated by the bar chart, which displays an enrichment factor (EF) of 27 for the top 3% ranking compounds. The model’s higher performance is quantitatively demonstrated by the conspicuous blue bar. The labeled value (EF = 27) shows that the technique finds 27 times more active compounds in this crucial early screening fraction than would be predicted by random selection (Fig 10A). This notable enrichment demonstrates the model’s remarkable capacity to effectively prioritize bioactive compounds, which is an important benefit for economical virtual screening campaigns because the early identification of genuine positives significantly lessens the experimental load.

Likewise, we constructed a virtual screening database for the MMP9 protein (PDB ID: 1GKC) that contained 12 experimentally verified active small compounds and 600 matching decoy molecules extracted from the DUD-E (Directory of Useful Decoys, Enhanced) database. Consequently, 612 compounds were included in the final screening library. The performance of the model is shown by the solid orange curve, whereas random categorization is shown by the dashed diagonal line. 0.85 (as seen in the legend), indicating that the model performed well. The enrichment factor for the top 3% of the rated compounds is shown in a bar chart. The enrichment factor is indicated by the blue bar, and the numerical value of 43 at the top shows the extent to which the model performs better than random selection. When compared to random selection, the model finds 43 times more actives in the top 3% of the predictions (Fig 10B).

To gather a comprehensive set of tiny compounds, we first carefully curated the dataset before broadening our search to include significant chemical databases such as ChEMBL, Mcule, PubChem, and ZINC [25,45,46,64]. A total of 1,500 intriguing compounds were found in our study’s additional research on both LCK and MMP9 proteins as a consequence of this thorough screening. This expanded dataset offers several opportunities for additional experimental assessments and enables strong confirmation of our computational technique.

### 3.7. ADMET analysis

A drug must have the best possible absorption, distribution, metabolism, excretion, and toxicity characteristics (ADMET) for commercial use. Therefore, in drug discovery, it is essential to accurately forecast these features. Using the SwissADME and ProTox-III web servers, we assessed the ADMET profiles of four ligands in this study: MCULE-1367186142, ZINC4896454, CID-56715853, and CID-126445459. The ADME analysis evaluated important parameters, including heavy atoms, aromatic heavy atoms, rotatable bonds, hydrogen bond acceptors and donors, Log Po/w, Log S (ESOL), gastrointestinal absorption (GI), blood-brain barrier (BBB) permeability, inhibition of cytochrome P450 enzymes (CYP1A2, CYP2C19, CYP2C9, CYP2D6, and synthetic accessibility. Furthermore, hepatotoxicity, carcinogenicity, immunotoxicity, mutagenicity, cytotoxicity, median lethal dose (LD50), and toxicity class were assessed using the ProTox-III toxicity profiling. The accompanying Table 4, provides a summary of the findings.

**Table 4:** ADME and toxicity features for four lead molecules.

Properties	MCULE-1367186142	ZINC4896454	CID-56715853	CID-126445459
ADME Features				
Physicochemical MW (g/*mol*)	415.51 g/*mol*	377.39 g/*mol*	362.38 g/*mol*	365.43 g/*mol*
Heavy Atoms	29	27	27	27
Aromatic Heavy Atoms	11	6	17	15
Rotatable Bonds	8	12	5	5
H-bond Acceptors	5	6	4	5
H-bond Donors	2	4	2	3
GLA	High	High	High	High
BBB	No	No	No	No
CYP1A2 Inhibitor	No	No	No	Yes
CYP2C19 Inhibitor	Yes	No	No	No
CYP2C9 Inhibitor	Yes	No	Yes	No
CYP2D6 Inhibitor	Yes	Yes	No	No
CYP3A4 Inhibitor	Yes	No	No	Yes
Lipinski	Yes	Yes	Yes	Yes
Synthetic Accessibility	3.82	2.8	2.83	3.55
**Toxicity Features**				
Hepatotoxicity	Inactive	Inactive	Inactive	Inactive
Carcinogenicity	Inactive	Inactive	Inactive	Inactive
Immunotoxicity	Inactive	Inactive	Inactive	Inactive
Mutagenicity	Inactive	Inactive	Active	Inactive
Cytotoxicity	Inactive	Inactive	Inactive	Inactive
LD50 (mg kg *−* 1)	1200	2025	489	1600
Toxicity Class	4	5	4	4

### 3.8. Molecular docking analysis

After evaluating the properties of ADMET, molecular coupling was performed using AutoDock Vina to estimate the binding affinities (kcal/mol) and interaction modes between the chosen ligands and target proteins (LCK, PDB ID: 1GKC; MMP9, PDB ID: 6PDJ). The docking settings were optimized, binding postures were examined, and important interaction data were extracted using a Python script.

Re-docking of the co-crystallized ligands into their appropriate binding sites allowed validation of the docking procedure. The reliability of the coupling approach was confirmed by the computed root-mean-square deviation (RMSD) values of 1.43 (1GKC) and 1.30 (6PDJ) between the expected and crystallographic positions.

#### 3.8.1. Interactions with LCK (PDB ID: 6PDJ).

**Fig 11:**
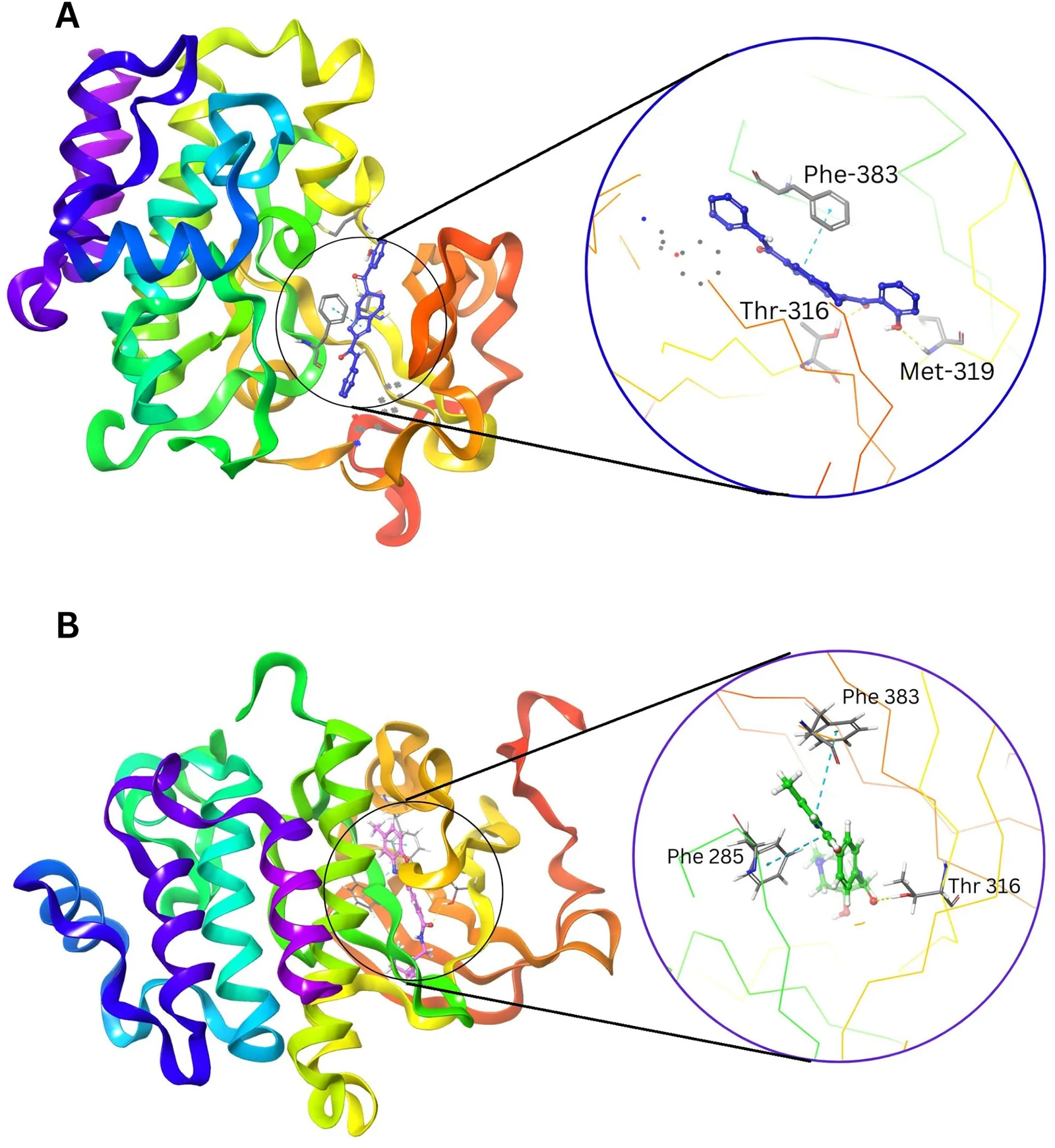
A) CID-56715853 and B) CID-126445459 Protein Ligand Complex with LCK(PDB ID:6PDJ).

Docking studies against LCK showed that both CID-56715853 and CID-126445459 formed stable complexes with the protein despite their differing binding affinities. Of all the ligands examined, CID-56715853 showed a binding affinity, of −9.7 kcal/mol. This strong link was made possible by hydrogen bond acceptors at Met 319 and in addition to hydrogen bond donors at Thr 316. Furthermore, *π*-*π* interactions with Phe 383 significantly increased the stability of the complex, indicating that CID-56715853 is a very desirable candidate for LCK inhibition. Furthermore,CID-126445459 showed a significant binding energy of −9.9 kcal/mol to LCK. This ligand produced significant hydrogen bond donors with Thr 316, although it exhibited more hydrogen bonding contacts than CID-56715853. Furthermore, similar to the CID-56715853 complex, *the π*-*π* interactions of Phe 383 were identified. These interactions suggested that CID-126445459 could be a good substitute for LCK (Fig 11A. CID-56715853 & B. CID-126445459).

#### 3.8.2. Interactions with MMP9 (PDB ID: 1GKC).

**Fig 12:**
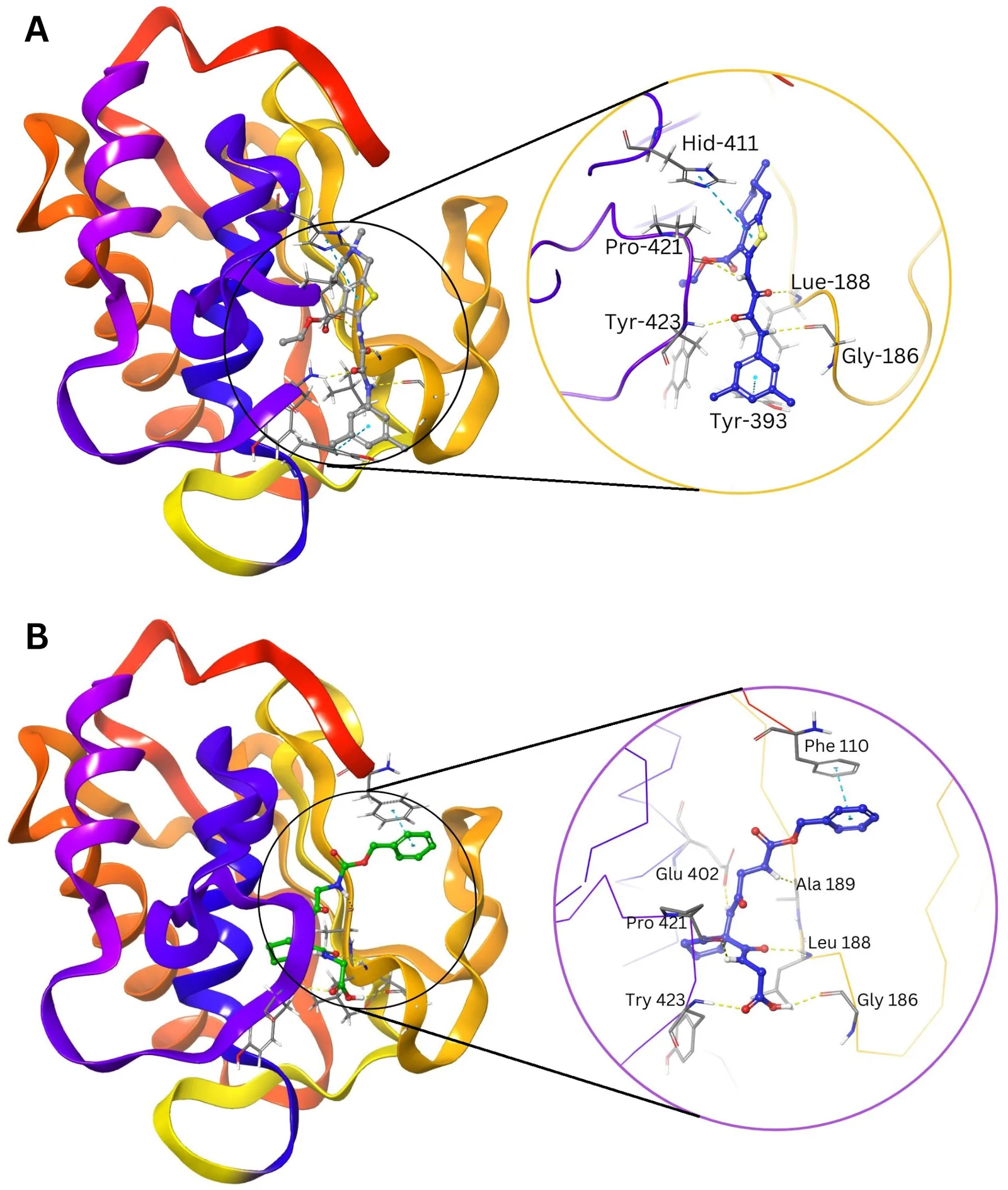
A) MCULE-1367186142 and B) ZINC4896454 Protein Ligand Complex with MMP9(PDB ID: 1GKC).

MCULE-1367186142 and ZINC4896454 had significant binding affinities and established stable complexes with the protein, as indicated by the docking data for MMP9. Having a binding value of −8.9 kcal/mol, MCULE-1367186142 had several significant interactions with the active site of the protein. Specifically, the ligand was utilized as an acceptor of hydrogen bonds with Tyr 423 and Leu 188 and a donor of hydrogen bonds with Pro 421 and Gly 186.*π*-*π* interactions between Tyr 393 and His 411 further solidified the ligand-protein combination. With an even higher binding affinity of −9.2 kcal/mol, ZINC4896454 demonstrated a more advantageous interaction with MMP9. This ligand donated hydrogen bonds to Pro 421, Gly 186, and Ala 189 while acting as an acceptor of hydrogen bonds with Leu 188, Glu 402, and Tyr 423. *π–π* stacking interactions with Phe 110 greatly improved the stability of the complex. These wide-ranging interactions indicated that ZINC4896454 has great potential as an MMP9 inhibitor (Fig 12. A MCULE-1367186142 & B. ZINC4896454).

Maestro 14.1 (Schr¨odinger) was used to view and evaluate protein-ligand interactions. Crucial binding residues are shown in the 3D interaction representations (Figs 10 and 11).

### 3.9. RMSD analysis

**Fig 13:**
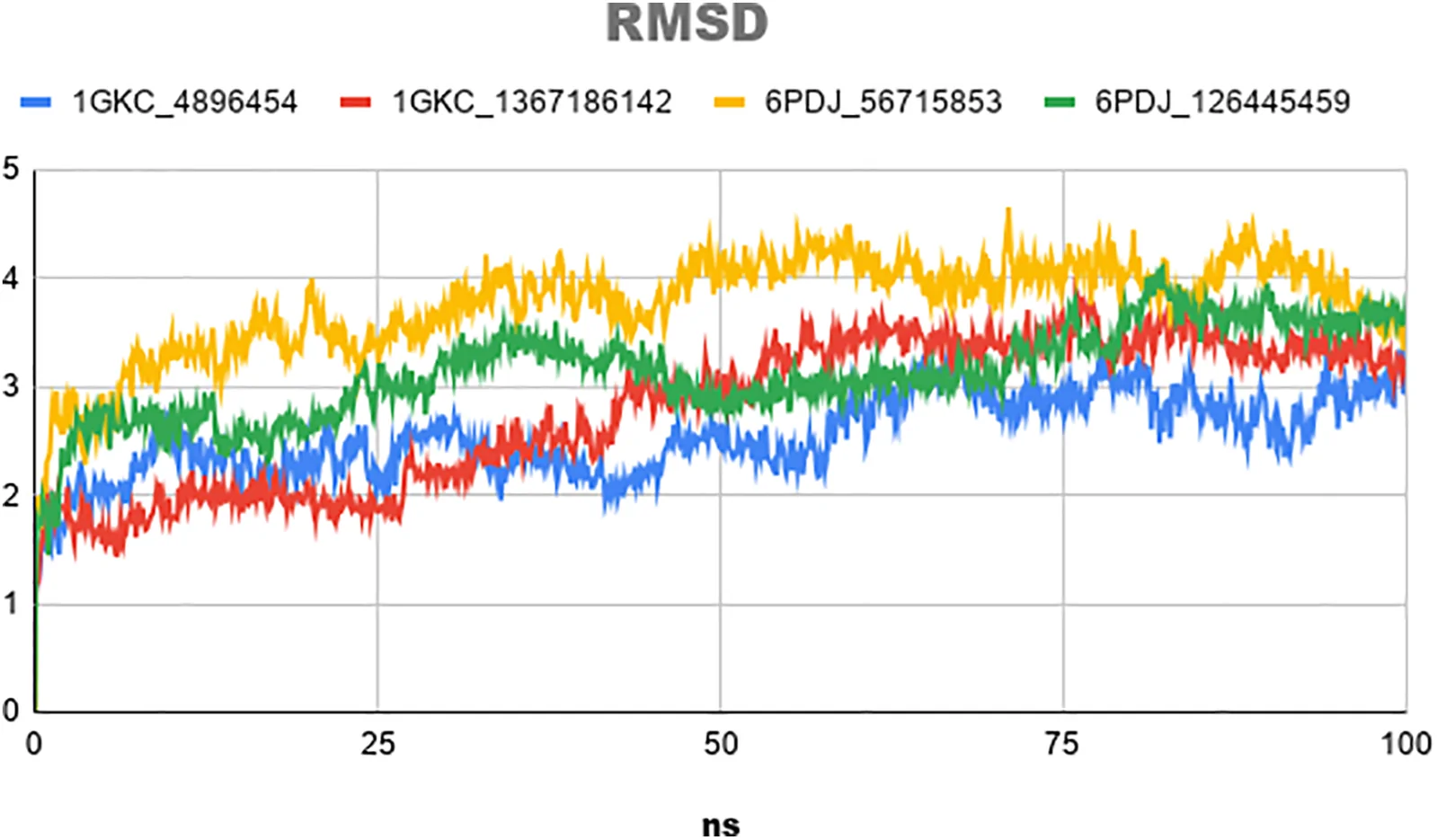
The RMSD values of the periodic progression of Four compounds.

Molecular dynamics simulation is a precise approach that can be used to evaluate and examine the motions of atoms underneath a system after a particular period of time has passed. The RMSD of protein (1GKC_4896454, 1GKC_1367186142, 6PDJ_56715853, and 6PDJ_126445459)C*α* atoms in the complexes are shown in Fig 13. From the RMSD Fig, it was clear that the 1GKC protein, when paired with the ligand ZNIC4896454, became stable from the initial stage and remained between 2 and 3 Å. From the moment the simulation started, the RMSD of the MCULE1367186142 ligand stabilized at approximately 10 ns and little bit near about 77 ns and remains within the range of 1.5 Angstrom and throughout the duration of the run at equilibrium [10].After reaching equilibrium with ligand CID56715853, the relative standard deviation (RMSD) of the 6PDJ protein was within the range of 0.5 to 1.5˚A. Additionally, protein 6PDJ exhibited slight fluctuations within the range of 2.0˚A during the course of the simulation, ranging from 40 ns to 60 ns but in CID126445459 ligand mode at 80 ns variance. The RMSDs of the protein structures (1GKC and 6PDJ) complexed with ZNIC4896454, MCULE1367186142, CID56715853, and CID126445459 increased at approximately the same rate, with almost negligible differences. In contrast to the other two ligands, compound 1GKC 4896454 stabilized at a higher value; its RMSD was also higher. RMSD remained steady up to 18 ns in instance 6PDJ 56715853, but then spiked from 2 to 3 Å, perhaps because the ligand mode slipped [32]. Mode flipping occurred at 30 ns in the 6PDJ 126445459 simulation; however, it stabilized after returning to balance.

### 3.10. RMSF analysis

**Fig 14:**
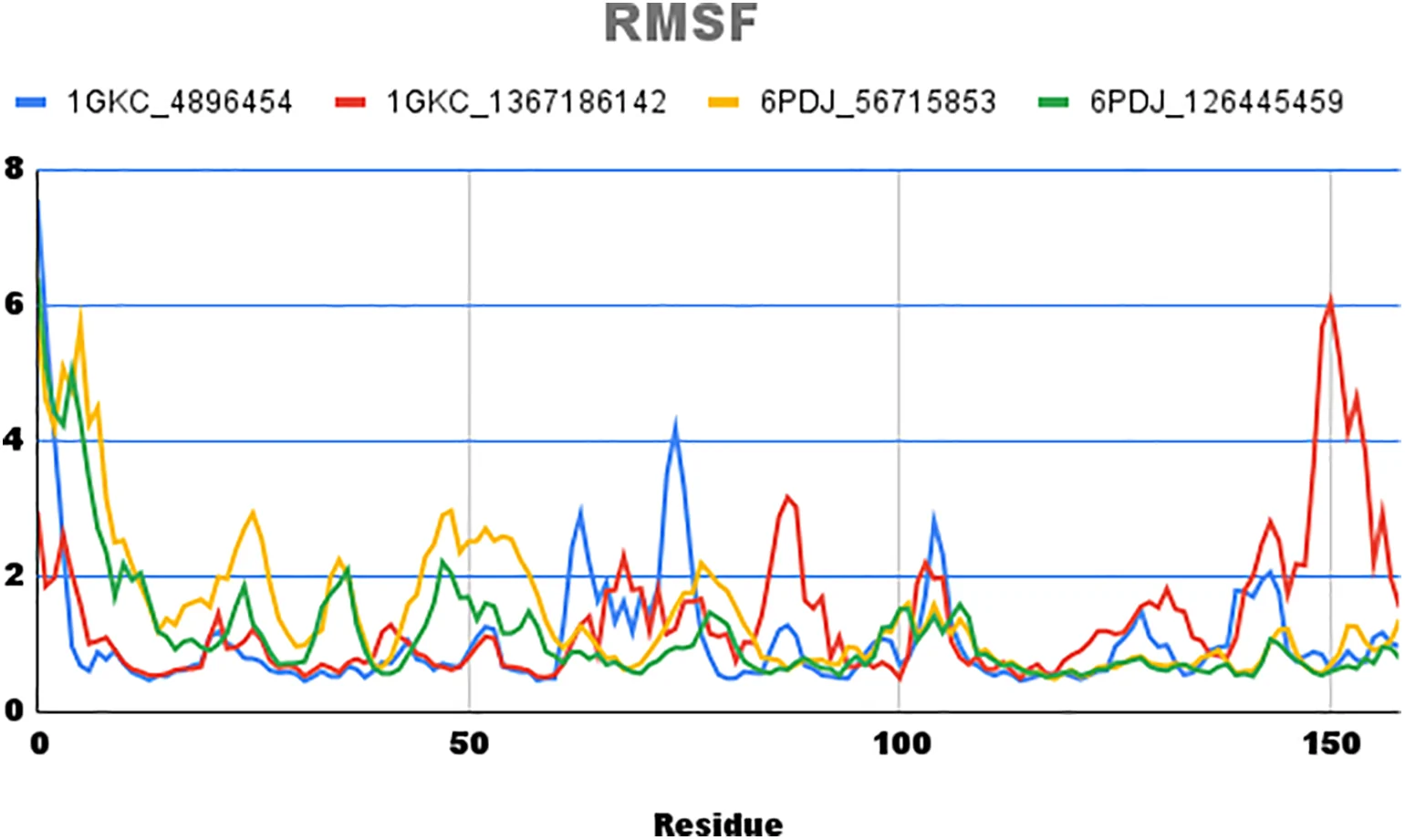
The RMSF values of the periodic progression of Four compounds.

Compared to RMSD, RMSF calculates the flexibility of individual residues or the amount by which a single residue varies in the simulation, as opposed to the positional variations between whole structures over time. It is common practice to plot the RMSF per residue against the number of residues, and this plot may provide structural information on which amino acids in a protein provide the most to molecular movement. The flexibility and changing dynamics of protein 1GKC and 6PDJ structures are shown in Fig 14, which provides information on these aspects. Most of the time, areas with high RMSF values are more flexible, while zones with low RMSF values are often more restricted. The fact that the RMSF values of the binding site residues were relatively low demonstrated that ligand binding to the protein was stable [76].

### 3.11. Protein-ligand contact analysis

**Fig 15:**
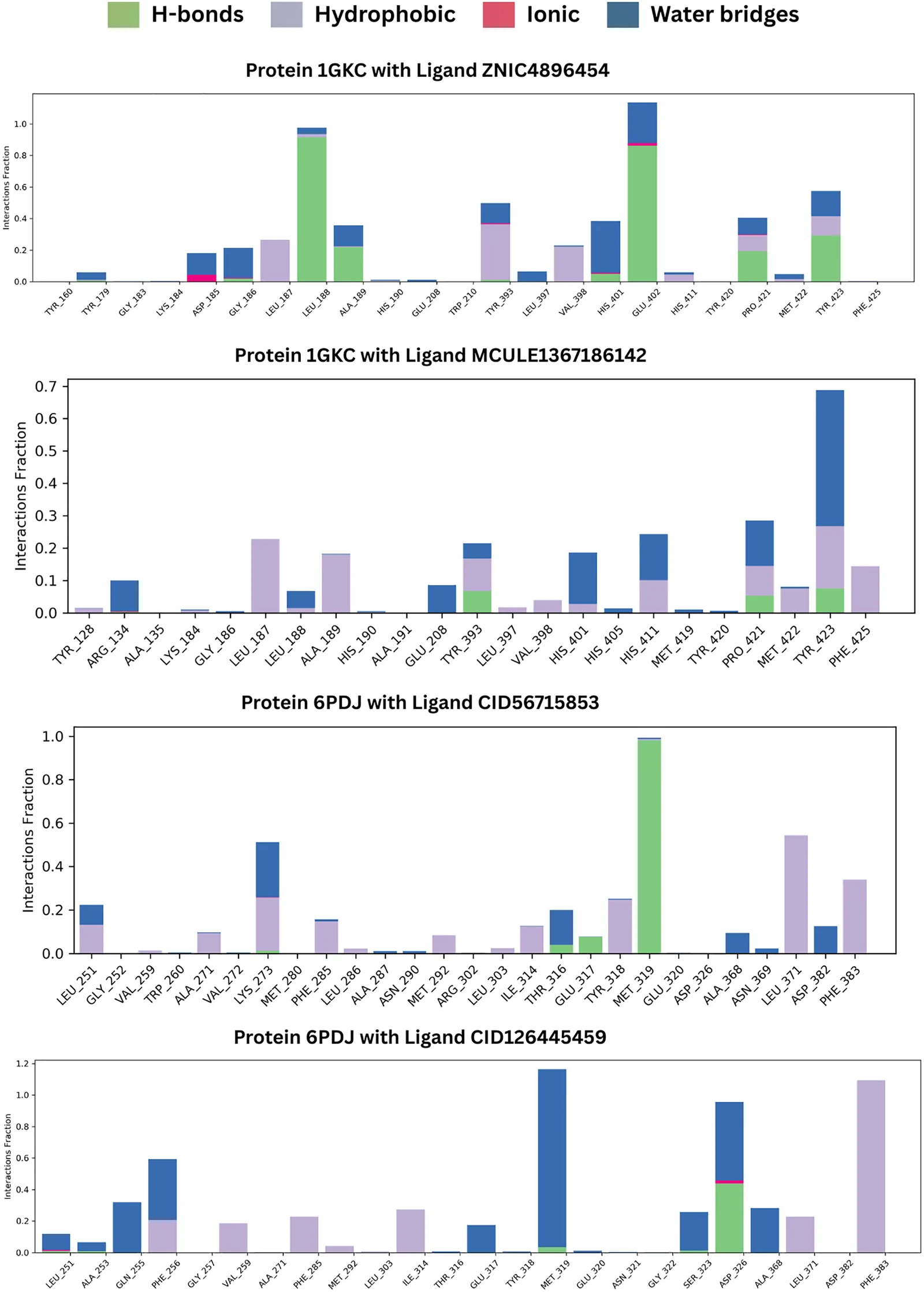
The Protein-Ligand Interactions Revealed During the 100 ns Simulation by the Stacked Bar Chart.

Simulation interaction diagrams were used to analyze the intricate structure of a protein, including its assigned ligands and the intermolecular interactions between them, over the course of a simulation period of 100 ns. On the basis of a numberof parameters, including hydrogen bonds, ionic bonds, non-covalent bonds (hydrophobic bonds), and water bridge bonds, the interaction between the protein and the chemicals ZNIC4896454, MCULE1367186142, CID56715853 and CID126445459 has been examined and illustrated; the results of this analysis are presented in Fig 15. During the 100 ns simulation, every chemical displayed a variety of interactions, which eventually resulted in the formation of a stable connection with the proteins of interest. The ranges of distinct bond types are shown in Fig 13. Residues ASP 185, GLY 186, LEU 188, ALA 189, TYR 393, HIS 401, GLU 402, and TYR 423 are especially crucial for hydrogen bonding in the 1GKC_4896454 complex. Significant correlations were found between the 1GKC_1367186142 complex and ARG 134, LEU 187, GLU 208, HIS 405, and TYR 423. LEU 251, LYS 273, PHE 285, ILE 314, MET 319, ALA 368, ASP 382, and PHE 383 in the complex 6PDJ_56715853. LEU 251,ALA 253,PHE 256,TYR 318,MET 319,ASP 382, and PHE 383 are important H-bonds in the 6PDJ_126445459 complex [15].

### 3.12. Principal Component Analysis(PCA)

After analyzing the changes and variations detected, three distinct principal components (PC1, PC2, and PC3) were identified. It is interesting to note that of all the PCs plotted for the four chosen complexes, PC3 had the least fluctuation, and a more compact structure was produced as a result of the reduced variability of PC3, which is also noticed as a more stable combination of the proteins. The ligands graph that was produced demonstrates that the blue color represents the most considerable mobility, the white color represents the intermediate movement, and the red color represents the least amount of flexibility [18].

**Fig 16:**
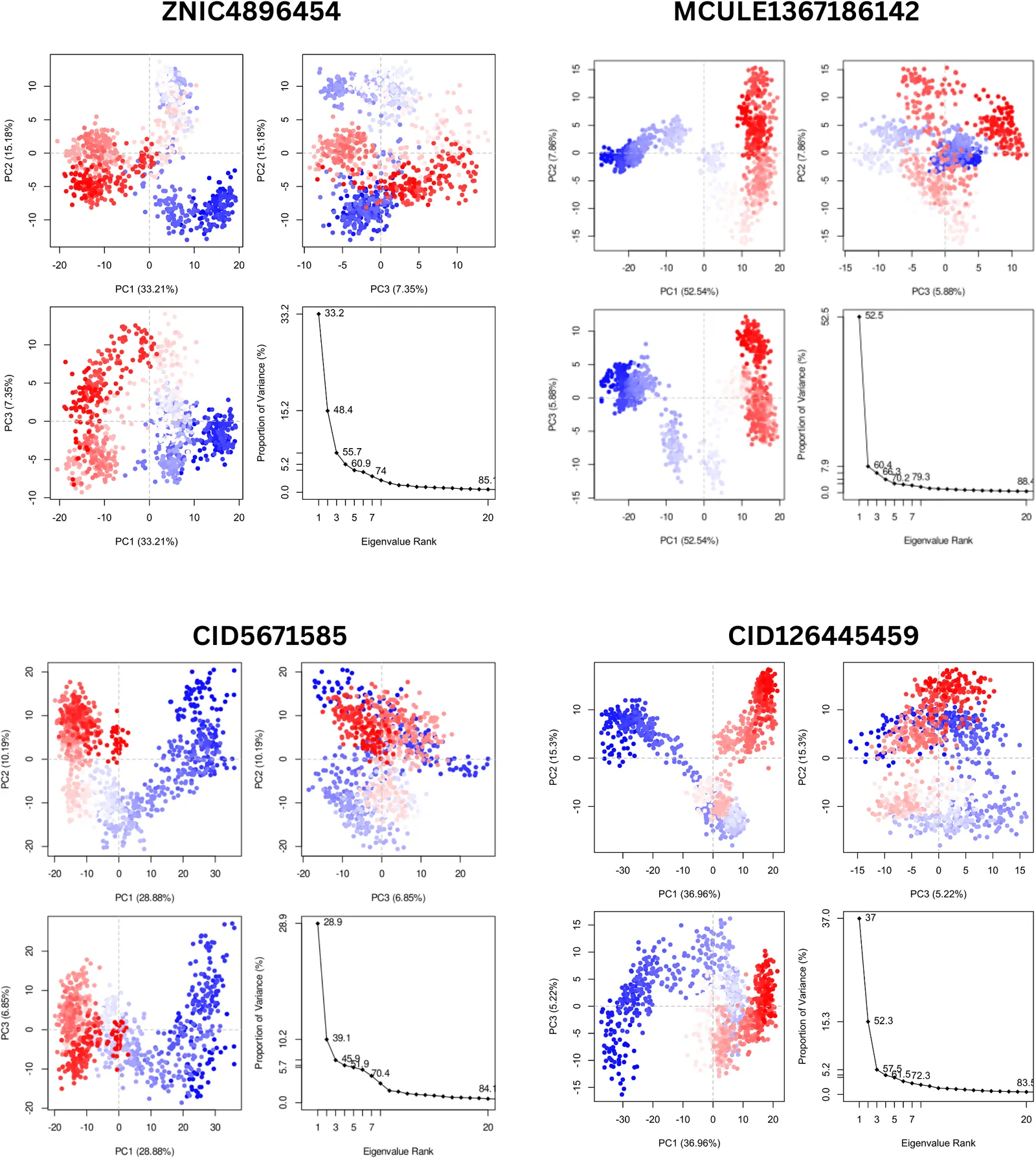
Eigenvalue in principal component analysis is a proportion of variance. three separate shows display the different areas(PC1,PC2,PC3).

PCA was used to describe the protein dynamics. For motion analysis, all graphs produced (Fig 16) of the protein (eigenvalues) versus the eigenmode demonstrated stability. The eigenvalues exhibit hyperspace eigenvector variations. Elevated eigenvalues were discovered for three of the chosen complexes, and the eigenvectors employed in the present systems exhibited dominant motions.

### 3.13. Dynamic Cross-Correlation Matrix Analysis(DCCM)

**Fig 17:**
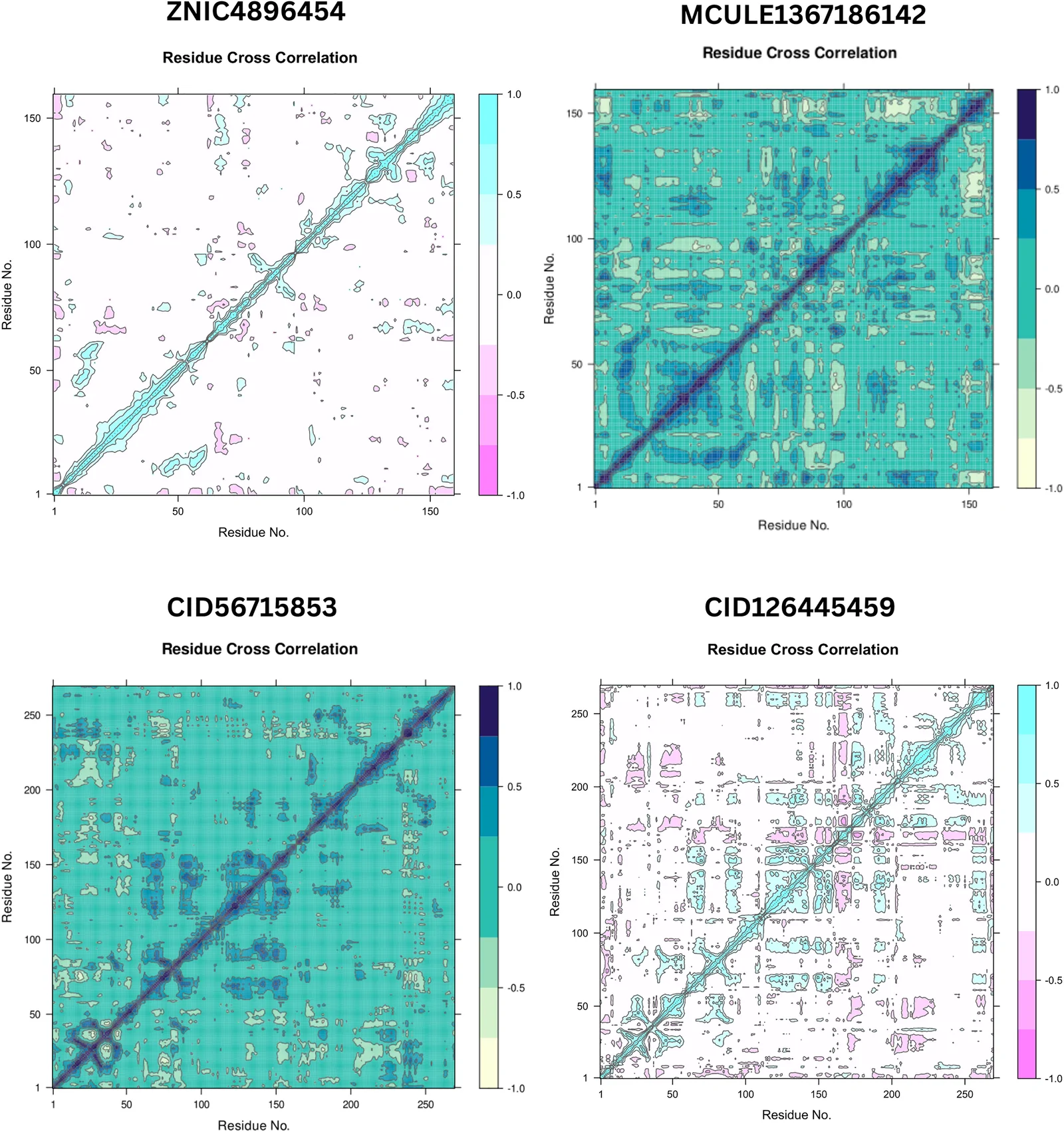
Detailed dynamic cross correlation maps.

For the motion analysis, all graphs produced (Fig 17) plot the eigenvalues of the protein against the eigenmode index, demonstrating global structural stability. The eigenvalue, which represents the stiffness or energy cost associated with each eigenmode, requires detailed explanation: lower eigenvalues correspond to large-scale, collective, and functionally relevant motions (soft modes), whereas higher eigenvalues indicate local, constrained vibrations (stiff modes). In this study, elevated eigenvalues were observed for three of the selected complexes, revealing that their corresponding eigenvectors — which describe the direction and magnitude of atomic displacement — exhibited dominant, cooperative motions [12]. Upon examination of the cross-correlation map, it was discovered that all three of the chosen complexes had an elevated pairwise cross-correlation coefficient importance, which indicated that the magenta colour was used to symbolize that they were substantially connected with one another. Anticorrelated sites (−0.4), whereas the cyan colour is used to convey correlated residues (*>* 0*.*8). Owing to the large number of pairwise correlated characteristics that exist between the 1GKC 4896454, 1GKC 1367186142, 6PDJ 56715853, and 6PDJ 126445459 proteins and their ligands, it is evident that the binding link between the two structures is persistent.

### 3.14. MM-GBSA analysis

In the process of determining the amount of energygands to bind to the 1gkcthe 1gkcthe 1gkcrecep-tors, MMGBSMMGBSMMGBSAedominant [30]. The binding free energies of the 1GKC 4896454, 1GKC1367186142, 6PDJ 56715853, and 6PDJ 126445459 complexes taken into consideration, as well as the consequences of additional non-bonded involvement energies, were considered. The binding energies of the ligands ZNIC4896454, MCULE1367186142, CID56715853, and CID126445459–1GKC and 6PDJ proteins were −70.85761925, −69.66328067, −76.11682902, and −80.73468743 kcal/mol, respectively Table 5. This resulted in MM-GBSA methods that emerged from the simulation trajectories, providing an appropriate validation for the binding energy derived from the docking data [27].

**Table 5:** Binding energy components for different protein-ligand complexes.

Energies (Kcal/mol)	1GKC 4896454	1GKC 1367186142	6PDJ 56715853	6PDJ 126445459
∆*G*bind	−70.85761925	−69.66328067	−76.11682902	−80.73468743
∆*G*Lipo	−29.93254934	−36.94305612	−34.86491179	−46.98557319
∆*G*vdW	−43.17185856	−47.79225297	−51.73811926	−52.99827831
∆*G*Hbond	−1.44659004	−0.172832135	−0.292817187	−0.094654210
∆*G*Packing	−0.00253475	−0.142696092	−0.314737373	−1.708044918

## 4. Discussion

Acute myeloid leukemia (AML) demonstrates substantial molecular disruptions, as evidenced by the identification of 566 differentially expressed genes (DEGs) compared to healthy controls. Of these, 437 genes were upregulated and 129 were downregulated, illustrating a pronounced shift toward oncogenic signaling and the suppression of tumor suppressor functions. These findings are consistent with the established knowledge of AML research [52]. Furthermore, the data underscores the involvement of immune-related pathways, such as cytokine signaling and apoptosis regulation, which highlights the critical role of immune system dysregulation in AML pathogenesis. Enrichment analyses, including KEGG and Reactome, identified key pathways, such as transcriptional misregulation in cancer” and “hematopoietic cell lineage,” both of which are widely recognized as central to AML’s molecular landscape of AML [52,56]. These pathways highlight the complexity of The study also identified ten hub genes within the protein–protein interaction (PPI) network: CD44, TNF, IL1B, MYC, STAT1, LCK, CCR7, FCGR3B, MMP9, and CD28. Several of these genes have been linked to leukemic cell survival, immune evasion, and proliferation. Notably, CD44 and MMP9 have established associations with metastasis and treatment resistance, whereas MYC and STAT1 are well-known oncogenic drivers in AMLL [17,37]. Further experimental validation of these gene candidates may facilitate the development of novel therapeutic strategies for AML treatment.

Pharmacophore models for LCK and MMP9 serve as highly efficient tools for structure-based virtual screening, streamlining the identification of potential inhibitors. In this study, models were derived from co-crystallized ligand–protein complexes, ensuring that key molecular interactions observed in experimental structures informed the model design [3]. For LCK, selectivity-driving features, such as hydrogen bonding (notably with Phe383 and Met318) and *π*-*π* stacking (Phe285, Phe383), were explicitly incorporated. The six-feature pharmacophore, comprising two hydrophobic, two aromatic, one hydrogen bond acceptor, and one donor group, reflects the established structural determinants of LCK inhibition [22]. Validation metrics further supported the model’s quality; ROC analysis returned an AUC of 0.79, and the enrichment factor was on par with the values reported in previous kinase-targeted pharmacophore studies. The MMP9 model prioritized Zn2 + coordination and hydrogen bonding (notably with Glu402, Pro421, and Tyr423), resulting in a five-feature pharmacophore (three acceptors and two donors) [55]. This model demonstrated strong performance with an AUC of 0.85 and an enrichment factor of 43 in the top 3%, underscoring its robustness even when explicit Zn2 + coordination was omitted due to modeling limitations. These findings are in line with prior research highlighting the significance of hydrogen-bond donors and acceptors in MMP9 inhibition [77]. The incorporation of molecular docking using AutoDock Vina provided an additional validation layer for evaluating both binding poses and energetics, thereby enhancing the reliability of the pharmacophore models. Overall, these models can significantly improve the efficiency of virtual screening by reducing false positives and computational demands, which is particularly advantageous in the early stages of drug discovery [69]. Thus, the LCK and MMP9 pharma-cophore frameworks developed in this study represent reliable platforms for the identification of novel inhibitors.

The evaluation of the four lead compounds, MCULE-1367186142, ZINC4896454, CID-56715853, and CID-126445459, highlights important pharmacokinetic and safety characteristics relevant to their therapeutic potential. All four exhibited high gastrointestinal absorption, indicating promising oral bioavailability. Notably, none of the compounds are predicted to cross the blood-brain barrier, which precludes central nervous system targeting but may also reduce the risk of CNS-related adverse effects. [6].Cytochrome P450 (CYP) inhibition patterns revealed that MCULE-1367186142 and CID-126445459 inhibited CYP1A2, CYP2D6, and CYP3A4, while CID-56715853 inhibited CYP2C19, CYP2C9, and CYP3A4. ZINC4896454 does not inhibit any major CYP enzymes, suggesting a favorable metabolic stability profile and a reduced risk of drug-drug interactions. [16]. All compounds conformed to Lipinski’s Rule of Five, supporting their drug-like properties. Synthetic accessibility scores are within a practical range, with CID-56715853 being the most readily synthesized, and MCULE-1367186142 being somewhat more challenging, although still feasible [48]. ZINC4896454 (2.80) and CID 56715853 (2.83) have similar synthetic accessibility ratings, suggesting that both compounds are easily synthesized. Synthetic accessibility scores range from 2.83 (CID-56715853, the simplest to synthesize) to 3.82 (MCULE-1367186142) [21].The toxicity profiles of the majority of the chemicals did not show discernible cytotoxicity, mutagenicity, immunotoxicity,or hepatotoxicity. However, there are safety concerns regarding the carcinogenic potential of CID-56715853. Low to moderate toxicity was indicated by the median lethal dose (LD50), which ranged from 489 mg/kg (CID-56715853, class 4) to 2025 mg/kg (ZINC4896454, class 5) [72]. In conclusion, ZINC4896454 and CID-126445459 showed encouraging safety and metabolic stability; however, the carcinogenicity of CID-56715853 and CYP inhibition of MCULE-1367186142 can limit their therapeutic use.

In summary, ZINC4896454 and CID-126445459 showed particularly favorable safety and metabolic stability profiles, making them strong candidates for further development. In contrast, MCULE-1367186142 CYP inhibition and the carcinogenicity concern associated with CID-56715853 may limit their therapeutic applications, and molecular docking studies have provided valuable insights into how the lead compounds interact with their target proteins, MMP9 (PDB: 6PDJ) and LCK (PDB: 1GKC). To ensure the reliability of these findings, a re-docking validation was performed, which confirmed the reproducibility of the docking protocol, as indicated by the low RMSD values of 1.30˚A for MMP9 and 1.43 ˚A for LCK [69]. For MMP9, both MCULE-1367186142 and ZINC4896454 demonstrated strong binding affinities, with docking scores of −8.9 kcal/mol and −9.2 kcal/mol, respectively. MCULE-1367186142 engaged in *π*-*π* stacking interactions with Tyr393 and His411 and formed hydrogen bonds with Tyr423 and Leu188 (as acceptors), as well as Pro421 and Gly186 (as donors). This pattern of interactions suggests a stable binding mode within the active site of MMP9. ZINC4896454 exhibited an even more extensive interaction network, forming hydrogen bonds via Pro421, Gly186, and Ala189 (as donors) and Leu188, Glu402, and Tyr423 (as acceptors), along with *π*-*π* stacking with Phe110. These results indicate that ZINC4896454’s enhanced interaction profile may translate to greater inhibitory activity against MMP9 [71].

In the case of LCK, CID-56715853 emerged as the strongest binder, with a docking score of −9.7 kcal/mol. Its key interactions include *π–π* stacking with Phe383 and hydrogen bonds involving Met319 (acceptor) and Thr316 (donor), which are likely to stabilize the ligand within the ATP-binding pocket of the kinase and contribute to its inhibitory potential [58]. CID-126445459 also maintained significant contacts, such as a hydrogen bond with Thr316 and *π*-*π* stacking with Phe283 and Phe383, although its binding affinity was somewhat reduced (−9.9 kcal/mol). This suggests that with further optimization, CID-126445459 could serve as a promising alternative LCK inhibitor. Overall, these findings highlight the potential of these compounds as effective inhibitors of their target genes.

Molecular dynamics simulations offered key insights into the stability and binding interactions of the protein-ligand complexes under investigation. Both the 1GKC-4896454 and 6PDJ-56715853 complexes reached equilibrium early, as indicated by their RMSD values within narrow ranges (2–3 A and 0.5–1.5 ˚A, respectively), suggesting robust and stable binding conformations. In contrast, the 6PDJ-126445459 complex exhibited some initial fluctuations before stabilizing at approximately 80 ns, likely reflecting early ligand adjustments prior to achieving an optimal binding pose [34]. Further support for the stability of these complexes comes from the RMSF analysis, which revealed limited mobility in residues around the binding site, which is consistent with established findings that stable binding often correlates with restricted residue movement in crystal regions [43].Protein-ligand contact analysis identified key residues involved in hydrogen bonding and hydrophobic interactions, both essential for maintaining complex stability and de-termining binding affinity and specificity [9]. Principal component analysis (PCA) and dynamic cross-correlation matrix (DCCM) analysis confirmed the structural integrity of the complexes, with minimal variation in the principal components and highly coordinated residue movements. This suggests that ligand binding does not induce significant conformational changes in the protein [18]. Moreover, MM-GBSA calculations indicated that the 6PDJ-126445459 complex exhibited the highest binding energy (–80.73 kcal/mol), primarily due to strong van der Waals and lipophilic contribu-tions—results that reinforce the central role of hydrophobic effects in molecular recognition. The good agreement between the docking and MD simulation results further strengthened the reliability of these findings [28]. Collectively, the simulations indicated that the studied complexes possessed favorable binding kinetics and stability, making them promising candidates for subsequent experimental validation.

We use an integrated computational method that integrates gene expression data, molecular docking, and molecular dynamics simulations to identify LCK and MMP9 as high-priority targets that need further investigation in AML. The body of in silico research suggests their potential diagnostic and therapeutic usefulness. Nonetheless, it is critical to acknowledge certain limitations of this research. First of all, the study was restricted to a single gene expression dataset, which may not fully capture the molecular heterogeneity of AML. Second, the computational drug discovery process that followed only targeted LCK and MMP9, even though our bioinformatics screening had originally found 10 hub genes. Thirdly, even though computational approaches offer insightful initial information, wet-lab tests are eventually required for experimental validation in drug discovery, which is outside the purview of this work. Future studies must address these constraints in order to move closer to more thorough and translational analyses of AML biomarkers.

## 5. Conclusion

Bioinformatics analysis of AML identified 566 differentially expressed genes and highlighted 10 hub genes central to disease pathogenesis. Pharmacophore models for LCK and MMP9 were developed and validated, enabling virtual screening for potential inhibitors. Four lead compounds demonstrated strong binding affinities, favourable ADMET profiles, and stable interactions through molecular docking and dynamics simulations. These findings provide a robust computational framework for prioritising novel biomarkers and drug candidates in AML, supporting further experimental validation and therapeutic development.
